# MALDI MSI Reveals the Spatial Distribution of Protein Markers in Tracheobronchial Lymph Nodes and Lung of Pigs after Respiratory Infection

**DOI:** 10.3390/molecules25235723

**Published:** 2020-12-03

**Authors:** Tomas Do, Roman Guran, Rea Jarosova, Petra Ondrackova, Zbysek Sladek, Martin Faldyna, Vojtech Adam, Ondrej Zitka

**Affiliations:** 1 Department of Chemistry and Biochemistry, Faculty of AgriSciences, Mendel University in Brno, 613 00 Brno, Czech Republic; xdo1@mendelu.cz (T.D.); roman.guran@mendelu.cz (R.G.); vojtech.adam@mendelu.cz (V.A.); 2 Central European Institute of Technology, Brno University of Technology, 612 00 Brno, Czech Republic; 3 Department of Morphology, Physiology and Animal Genetics, Faculty of AgriSciences, Mendel University in Brno, 613 00 Brno, Czech Republic; rea.jarosova@mendelu.cz (R.J.); zbysek.sladek@mendelu.cz (Z.S.); 4 Department of Immunology, Veterinary Research Institute, 621 00 Brno, Czech Republic; ondrackovap@vri.cz (P.O.); faldyna@vri.cz (M.F.); 5 Central European Institute of Technology, Mendel University in Brno, 613 00 Brno, Czech Republic

**Keywords:** CD163, interleukin 1β, protegrin-4 precursor, MALDI MSI, pig model, lungs infection, *Actinobacillus pleuropneumoniae*

## Abstract

Respiratory infections are a real threat for humans, and therefore the pig model is of interest for studies. As one of a case for studies, *Actinobacillus pleuropneumoniae* (APP) caused infections and still worries many pig breeders around the world. To better understand the influence of pathogenic effect of APP on a respiratory system—lungs and tracheobronchial lymph nodes (TBLN), we aimed to employ matrix-assisted laser desorption/ionization time-of-flight mass spectrometry imaging (MALDI-TOF MSI). In this study, six pigs were intranasally infected by APP and two were used as non-infected control, and 48 cryosections have been obtained. MALDI-TOF MSI and immunohistochemistry (IHC) were used to study spatial distribution of infectious markers, especially interleukins, in cryosections of porcine tissues of lungs (necrotic area, marginal zone) and tracheobronchial lymph nodes (TBLN) from pigs infected by APP. CD163, interleukin 1β (IL-1β) and a protegrin-4 precursor were successfully detected based on their tryptic fragments. CD163 and IL-1β were confirmed also by IHC. The protegrin-4 precursor was identified by MALDI-TOF/TOF directly on the tissue cryosections. CD163, IL-1β and protegrin-4 precursor were all significantly (*p* < 0.001) more expressed in necrotic areas of lungs infected by APP than in marginal zone, TBLN and in control lungs.

## 1. Introduction

*Actinobacillus pleuropneumoniae* (APP) is a common bacterial agent, belonging to the *Pasteurellaceae* family, causing swine contagious pleuropneumonia, which belongs to the ordinary swine respiratory disease and highly influences the incomes of pig breeders. To date, there exist 2 biovars and 18 serovars of APP*,* which have different virulence [1]. The virulence of APP is dependent on factors like a biofilm formation, production of exotoxins (Apx) or pore-forming repeats-in-toxin exoproteins and an antimicrobial resistance [2,3]. As main virulence factors are deemed ApxI-III toxins [4]. The innate production of monocyte/macrophage specific markers like CD163 and the production of pro-inflammatory cytokines (IL-1β, IL-6, IL-17, TNF-alpha and others) is a sign of a typical immune response of organism against APP [4,5,6]. In addition, CD8-negative γδ-TCR T cells and Th17 cells (CD4+CD8αdimIL-17A+) are linked to immune responses against APP, too [4,7,8]. For a detection and characterization of interleukins and other infectious markers connected to APP infection, the in vitro model of porcine immortalized epithelial cell lines can be used [9]. The virulence of *A. pleuropneumoniae* can be attenuated by creating double-deletion DNA mutants with the purpose of obtaining possible vaccines [10,11].

The APP infection in porcine lungs and other organs can be studied by various methods, such as: PCR/cDNA microarrays [12], flow cytometry [5,8,13], ELISA [8,14,15], histology [14,16] and immunohistochemistry (IHC) [17]. To the best of our knowledge, regarding the mass spectrometry imaging (MSI), only two techniques were used so far to study porcine infection by APP: nanoscale secondary ion mass spectrometry (nanoSIMS) and matrix-assisted laser desorption/ionization time-of-flight mass spectrometry imaging (MALDI-TOF MSI). NanoSIMS was used for APP cells [10], whereas our group used MALDI-TOF MSI for imaging cryosections of porcine lungs and other tissues [18,19].

The classical approach, when spatial information about various biological processes in tissues is required, uses the IHC, in situ hybridization or similar techniques. These techniques need a knowledge of the targeted analyte prior the analysis and only few analytes can be analyzed simultaneously during one run of the analysis. Moreover, these methods often lack specificity for molecules such as drugs and their metabolites, lipids, protein isoforms, and mutations, post-translational modifications, and products of protein cleavage [20].

MALDI-TOF MSI belongs to label-free imaging methods and is used to detect and identify, if possible, different (bio-)molecules in tissue sections and to map their spatial distribution in these sections [21,22]. This method can be used to support histology/IHC and vice versa [22]. It is worth to mention that mass spectrometry imaging, in general, makes possible the detection/identification of (bio-)molecules for which the IHC antibodies are not available [23], as it is the case for protegrin-4 precursor in this study, or for which other imaging methods are not suitable. MALDI-TOF MSI is commonly applied for a detection/identification of smaller proteins (usually with molecular weights not exceeding 30 kDa) [18,24], peptides [25,26], lipids [27], oligosaccharides [28], etc. [29]. It was successfully used in a research of various tumors and infections [30,31,32].

In this study, we have focused on the possibilities of a MALDI-TOF MSI for a detection and identification of CD163, interleukins and other infectious markers in cryosections of porcine lungs and other closely connected tissues in pigs infected by APP. We wanted to show the possible new markers of porcine lungs infection and if they correlate with interleukin and/CD163. In addition, we wanted to show the capability of MALDI-TOF MSI and its use for detection of analytes which do not have IHC antibodies.

## 2. Results and Discussion

During the development and optimization of our MALDI-TOF MSI method we were able to map distribution of cell markers of lymphocytes, monocytes and/or macrophages in porcine tissue cryosections infected by APP [18]. This achievement followed a recent publication by Holzechner et al. [33], who used MALDI-TOF MSI to visualize the localization of lymphocytes and macrophages in human colon tissue sections.

In the present study, we have focused on detecting, identifying, and mapping a typical inflammation marker CD163 [34], and specific interleukins and other infectious markers in cryosections of porcine lung tissue affected by APP. After on-tissue trypsinization, we searched MALDI MSI data for specific peptides of CD163 and different interleukins (IL-1β; IL-6; IL-8). The sequences of specific peptides were determined from protein sequences (downloaded from UniProt database) after in silico digestion with trypsin using SequenceEditor (Bruker Daltonik GmbH, Bremen, Germany). The experimental data, corresponding to in silico data, matched with peptides of CD163 and IL-1β. The CD163 tryptic fragment/peptide TSYQVYSK was represented by a mass peak at *m*/*z* 975.478 ± 0.100 Da. The IL-1β tryptic fragment/peptide NLYLSCVLKDDKPTLQLESVDPKNYPK was represented by a mass peak at *m*/*z* 3119.621 ± 0.100 Da. To support information from MALDI MSI, an IHC analysis for CD163 and IL-1β of consecutive cryosections was performed. We have demonstrated some preliminary data for the IL-1β tryptic peptide in our previous work [19]. In the present study, we have used different consecutive and other cryosections for MALDI MSI and IHC analysis. Immunohistochemistry (IHC) is a classical technique to study protein localization in tissues, as well as to confirm a protein’s identity, and it is therefore complimentary to MALDI MSI studies [35]. IHC analysis was used in many protein or cancer studies as support method for MALDI MSI such as studies of prostate tumor [23,36], skin tumors [31,32], head and neck tumor [37] or non-small cell lung tumor [38].

**Figure 1:**
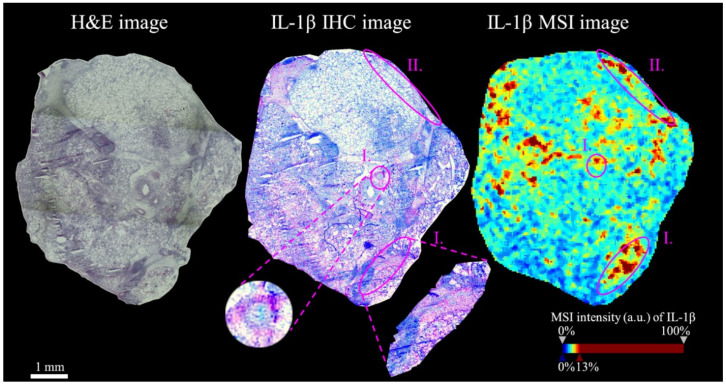
Spatial distribution of IL-1β peptide on one zoomed cryosection of lung tissue from pig infected by *Actinobacillus pleuropneumoniae* (APP)—necrotic area (NA). The matrix-assisted laser desorption/ionization time-of-flight mass spectrometry imaging (MALDI-TOF MSI) detection of IL-1β peptide NLYLSCVLKDDKPTLQLESVDPKNYPK (3119.621 ± 0.100 Da) was confirmed by comparison with immunohistochemistry (IHC) of IL-1β on consecutive tissue cryosection. The purple ellipses are highlighting regions with detected IL-1β. The regions I. show MSI signal corresponding with IHC signal (light purple). The region II. shows only MSI signal, no IHC signal was detected. The zoomed mean mass spectrum of IL-1β peptide is presented in Figure S18.

In Figure 1 is presented one zoomed cryosection to provide more detailed information on spatial distribution. The regions I. (Figure 1, highlighted by purple ellipses) are showing the correspondence of IHC positive signal (brown color) for IL-1β with MSI data of IL-1β tryptic fragment (see the Figure S18 for zoomed mean mass spectrum of IL-1β peptide). The IL-1β tryptic fragment was detected also in regions, where IHC showed no or small signal, as it is demonstrated in region II. (Figure 1; highlighted by purple ellipses).

**Figure 2:**
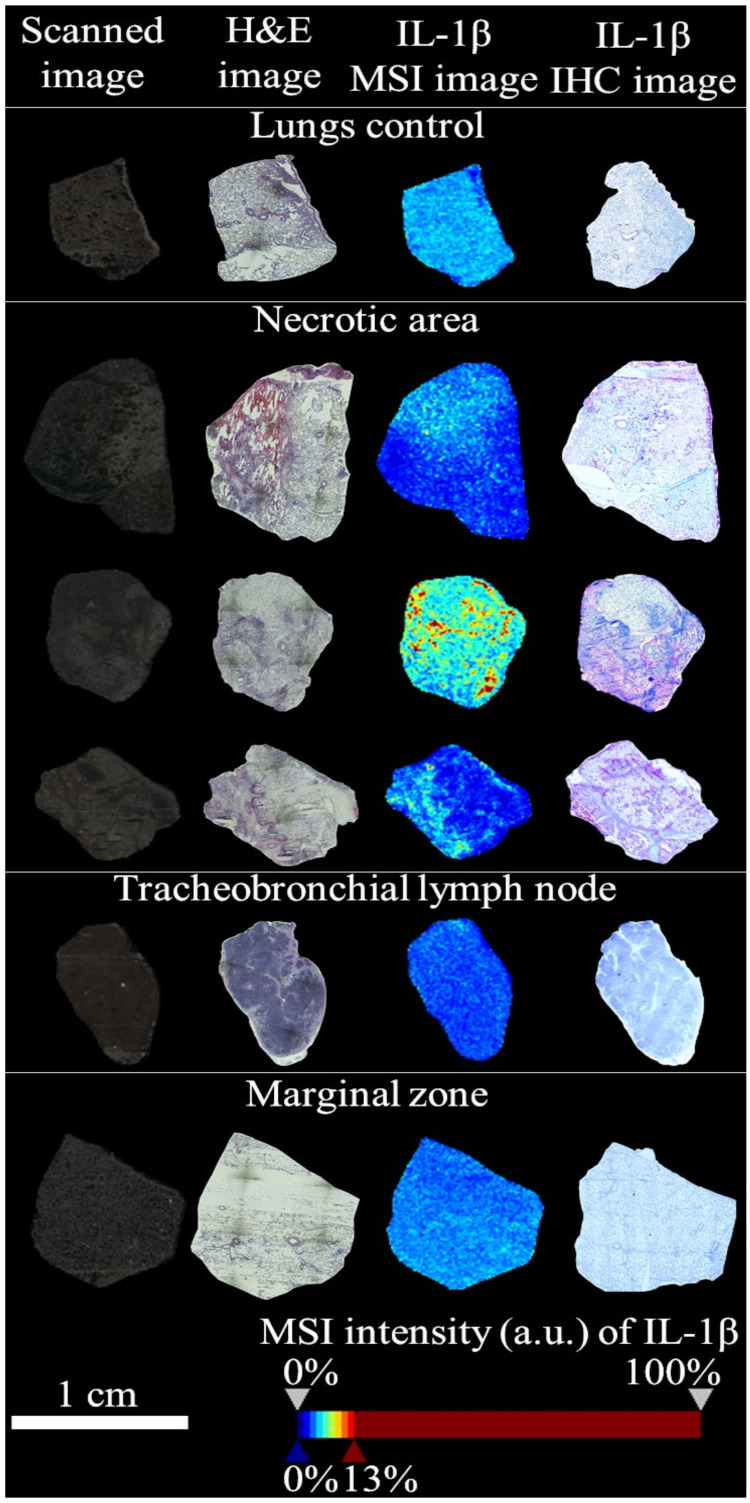
Spatial distribution of IL-1β peptide on porcine tissue sections of control lungs and necrotic area (NA), marginal zone (MZ) and tracheobronchial lymph nodes (TBLN) from pigs infected by APP. For MALDI-TOF MSI images, the IL-1β peptide NLYLSCVLKDDKPTLQLESVDPKNYPK (3119.621 ± 0.100 Da) was detected. The representative images were used. The more detailed IHC images before and after their modification in GIMP 2.10.22 are presented in Supplementary Data as Figures S1–S6. The zoomed mean mass spectrum of IL-1β peptide is presented in Figure S18.

Figure 2 shows representative MSI and IHC data for IL-1β detected in more porcine tissue cryosections of control lungs, lungs infected by APP—necrotic area (NA) and marginal zone (MZ), tracheobronchial lymph nodes (TBLN). The more detailed representative IHC images, before and after their modification in GIMP 2.10.22, of control lung (Figure S1), NA (Figures S2–S4), TBLN (Figure S5) and MZ (Figure S6) are provided in the Supplementary Data. The MSI data were detected IL-1β tryptic fragment even in the positions where IHC antibody anti-IL-1β was not bound properly.

We have also tried to identify infectious markers directly on a surface of porcine lung cryosections by using MALDI-LIFT-TOF/TOF for selected highly intensive mass peaks of tryptic peptides. We were successful to identify some normally present proteins like hemoglobin (data not shown), but more importantly, even a Protegrin-4 precursor, belonging among the porcine leukocyte-derived protegrins, which have antimicrobial properties. Expression of these antimicrobial peptides supports the innate immune response and prevent from further tissue damage [39]. For the results from MASCOT search, see Figure S13. The annotated LIFT fragment spectrum of Protegrin-4 precursor peptide is presented in Figure S14, the sequence coverage of whole Protegrin-4 precursor is presented in Figure S15 and LIFT spectrum analysis report is presented in Figure S16. The obtained sequence coverage is low (4%), but the identified peptide is a unique peptide for the Protegrin-4 protein family (according to MASCOT search results in Figure S13). The zoomed mean mass spectrum of Protegrin-4 precursor peptide is presented in Figure S19.

**Figure 3:**
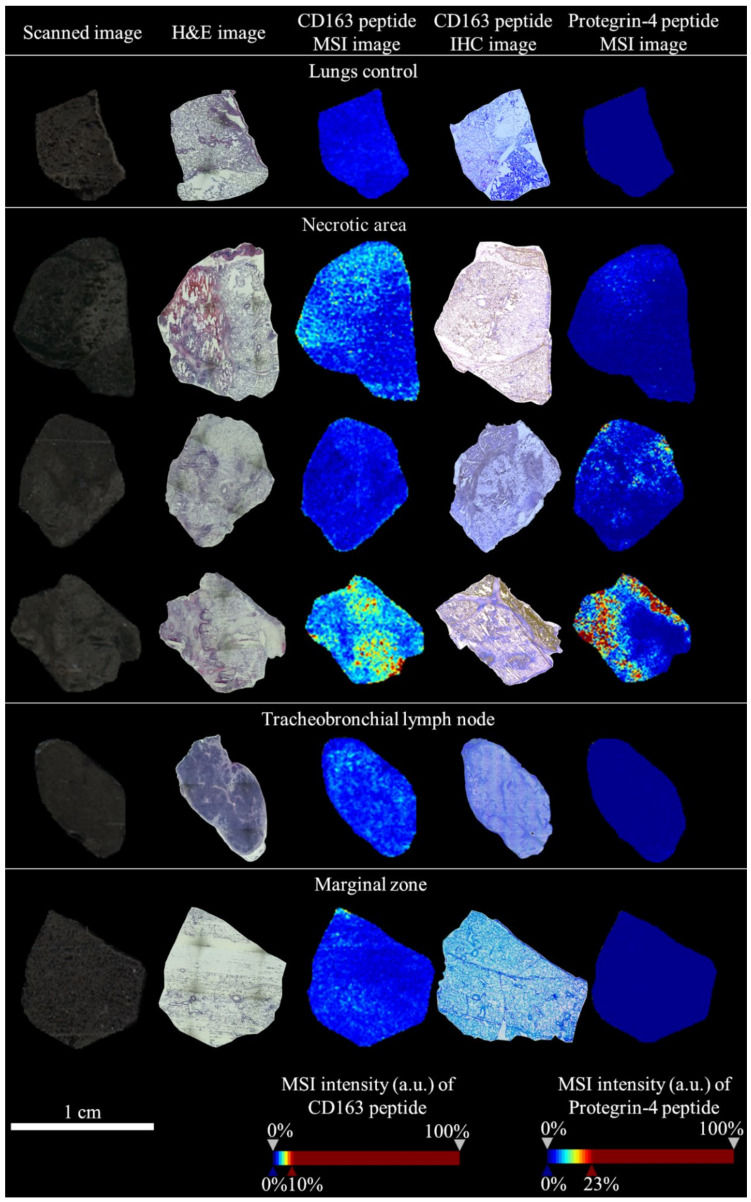
Spatial distribution of CD163 peptide and Protegrin-4 precursor peptide on porcine tissue sections of control lungs and NA, MZ and TBMU from pigs infected by APP. For MALDI-TOF MSI images, the CD163 peptide TSYQVYSK (975.478 ± 0.100 Da) and Protegrin-4 precursor peptide FPPPNFPGPR (1126.216 ± 0.100 Da) were detected. The Protegrin-4 precursor protein was identified by on-tissue MALDI-LIFT-TOF/TOF of his peptide FPPPNFPGPR and by using in-house MASCOT Server. The MASCOT score was 54 (see more info in Figure S13). The representative images were used. The more detailed IHC images before and after their modification in GIMP 2.10.22 are presented in Supplementary Data as Figures S7–S12. The zoomed mean mass spectrum of protegrin-4 precursor peptide is presented in Figure S19 and of CD163 peptide in Figure S20.

There are no antibodies for IHC of Protegrin-4 precursor (according to our knowledge), so we have used only MSI data to show the spatial distribution of Protegrin-4 precursor (Figure 3). The Protegrin-4 precursor tryptic fragment/peptide FPPPNFPGPR, represented by a mass peak at *m*/*z* 1126.216 ± 0.100 Da, was used to visualize the distribution. Figure 3 also shows representative MSI and IHC data for CD163 detected in porcine tissue cryosections of control lungs, lungs infected by APP—necrotic area (NA) and marginal zone (MZ), tracheobronchial lymph nodes (TBLN). The zoomed mean mass spectrum of CD163 peptide is presented in Figure S20.

**Figure 4:**
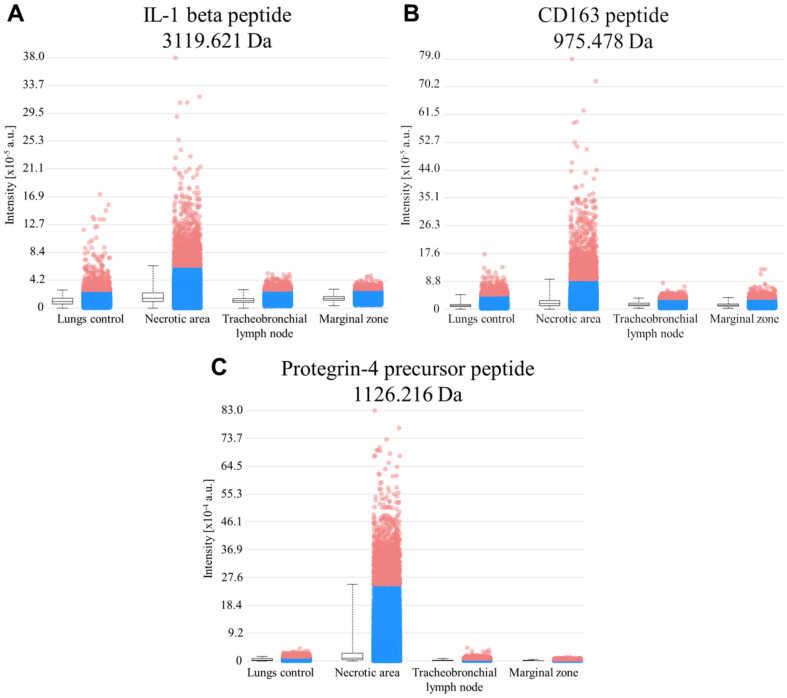
Intensity box plots for (**A**) IL-1β peptide NLYLSCVLKDDKPTLQLESVDPKNYPK (3119.621 ± 0.100 Da), (**B**) CD163 peptide TSYQVYSK (975.478 ± 0.100 Da) and (**C**) Protegrin-4 precursor peptide FPPPNFPGPR (1126.216 ± 0.100 Da) showing statistically significant (*p* < 0.001) differences between lungs infected by APP—NA and other samples, including control lungs, MZ and TBLN. The number of measured cryosections used for statistical analysis was 12 cryosections per each sample type.

The intensity box plots from MALDI MSI data for IL-1β tryptic peptide (Figure 4A), CD163 tryptic peptide (Figure 4B) and for Protegrin-4 precursor tryptic peptide (Figure 4C), together with Kruskal–Wallis test (Table 1), revealed, that all peptides were significantly (*p* < 0.001) more expressed in porcine tissue cryosections of lungs infected by APP—NA, than in other cryosections like control lungs, MZ and TBLN. Therefore, all these peptides could be used as the possible infection markers. The results from MALDI MSI are in a good correlation with IHC data for IL-1β and CD163.

Regarding IL-1β, its connection to inflammation caused (not only) by APP is well known [40,41], but there are no publications on Web of Science, at the time of writing this article, after using searching words “interleukin AND mass spectrometry imaging AND pig”, dealing with detection of IL-1β by mass spectrometry imaging in porcine tissue samples. Additionally, for protegrin-4 precursor, there exist some connections of protegrin-like antimicrobial peptide with APP infection [42] but, at the time of writing this article, after using searching words “protegrin AND mass spectrometry imaging”, there are no publications on Web of Science devoted to protegrin-4 precursor detection by mass spectrometry imaging.

Based on MSI images of IL-1β peptide (Figure 2) and protegrin-4 precursor peptide (Figure 3) in necrotic area (NA), both peptides showed co-detection. We can assume that IL-1B and protegrin-4 are connected and that they can have similar effect, possibly also synergistic effect. For that there is no direct evidence in literature on Web of Science, but there exists a connection of protegrins 1 and 3 with initiation of IL-1B posttranslational processing described by Perregaux et al. [43]. It is probable, that protegrin 4 acts similarly. In terms of the therapy of the swines, the detection itself of the presence of protegrin-4 precursor in NA must be considered as a positive result due to the existence of antibiotic-resistent *A. pleuropneumoniae* strains [44] or other multidrug-resistent patogens. Antibiotic resistance is a global problem, and protegrins as therapeutic agent are considered as a possible solution and a replacement of conventional antibiotics [45].

Regarding the CD163 protein, there is no direct connection with the expression of protegrins described in the literature, but the connection of CD163 protein and expression of interleukins is described well [46,47,48].

The need of detecting cytokines and other infectious markers in different tissues, mainly human, is getting more attention in past months especially because of the COVID-19 pandemic and its connection to cytokine storm caused by the SARS-CoV-2 [49,50,51]. Currently, increased level of IL-6 in patients infected with COVID-19 and its correlation with the disease mortality is a very discussed issue [52]. By using direct analytical approaches, as is in this case a mass spectrometry imaging, it may be possible to react to the need of detecting spatial distribution of interleukins and other molecules connected to the inflammatory response of the organism quicker than by using indirect analytical methods like IHC—especially if the IHC-antibody for the targeted molecule has not been developed yet. In addition, based on comparison between IHC and MSI images, MALDI MSI is able to display the occurrence of the infectious markers in the specific regions of the cryosections where IHC method is not able to detect the presence of the selected markers. This knowledge could be potentially beneficial in future clinical studying in the field of a targeted therapy. Of course, the mass spectrometry imaging is not an in vivo method, nevertheless it can provide more information about the inflammation and/or other processes in organism.

## 3. Materials and Methods

### 3.1. Chemicals and Materials

The MALDI matrix 2,5-dihydroxybenzoic acid (DHB) and other chemicals/material were ordered in suitable quality from Sigma-Aldrich (St. Louis, MO, USA) if not otherwise noted. Conductive indium-tin oxide (ITO) glass slides and peptide calibration standards were purchased from Bruker Daltonik GmbH (Bremen, Germany).

### 3.2. Sample Collection

For the study, pigs of Large White breed of 28 days old originated from herd at experimental accredited stables of Veterinary Research Institute (Brno, Czech Republic), with a good epidemiological situation, were used. The experiment was approved by the Branch Commission for Animal Welfare of the Ministry of Agriculture of the Czech Republic (approval no. 31674/2018-MZE-17214) and complied with the Act No. 246/1992 Coll. of the Czech National Council on the protection of animals against cruelty. Pigs were negatively tested for a presence of APP-specific antibodies. The total number of pigs includes six individuals infected by APP and two individuals as non-infected control. The infection of pigs with infectious dose of 2 × 10^9^ APP (field-origin strain, biotype 1, serotype 9, KL2-2000), the collection of pulmonary tissue samples after euthanasia—specifically necrotic area of lungs (NA), area between necrosis and visually unaffected area—marginal zone (MZ) and tracheobronchial lymph nodes (TBLN), and the preparation of cryosections onto ITO glass slides for MALDI MSI was performed according to Jarosova et al. [19]: The infection of pigs with the infectious dose of 2 × 10^9^ APP (field-origin strain, biotype 1, serotype 9, KL2-2000) was performed by administration of the infectious bacterium to the second third of each nasal cavity intranasally during inhalation. 

Immediately after euthanasia, samples of pulmonary tissue were acquired. Lung tissue was sampled from necrotic areas (NA), from visually unaffected areas (UA) and from areas bordering on necrotic areas—marginal zone (MZ). Samples of lungs were filled with mixture of cryoprotective embedding medium Tissue Tek (O.C.T. Compound, Sakura-Finetek, Tokyo, Japan) (OCT) with phosphate buffered saline (Bio Whittaker, Lonza, Basel, Switzerland) (PBS) in ratio 1:1 for preserved open alveolar structure, embedded in OCT and frozen by supercooled n-heptane placed on dry ice.

The tissue samples were cut to a thickness of 10 µm on the cryostat (Leica Microsystems, CM 1900, GmbH, Wetzlar, Germany) at temperature −20 °C. The cuts of tissue were placed on ITO slides, the sections were allowed to dry at room temperature, fixed in pre-cooled acetone (PENTA s.r.o., Praha, Czech Republic) at −18 °C for 5 min and stored at −80 °C.

### 3.3. Preparation of Cryosections for MALDI-TOF MSI

The preparation of cryosections for MALDI matrix application and on-tissue trypsinization followed the protocol of Jarosova et al. [19]: The ITO glass slides were warmed by a hand at room temperature to thaw-mount the cryosections and desiccated under vacuum for 15 min at a Vacufuge Concentrator (Eppendorf, CZ). Then, the slides were washed in a glass Coplin jar with 70% ethanol for 2 min twice and 100% ethanol for 2 min once. In the next steps prior the extraction of peptides, samples were desiccated under vacuum for 15 min, after that three guide marks using a white pencil corrector were marked for determining positions of cryosections in FlexImaging 3.0 software (Bruker Daltonik GmbH, Bremen, Germany). Afterwards, images of the ITO glass slides at a resolution of 3200 DPI were taken by an Epson Perfection V500 Office scanner (Epson Europe B.V., Amsterdam, The Netherlands).

On-tissue trypsinization: Trypsin solution (0.075 μg/μL) was prepared by reconstituting trypsin powder (20 µg) with 40 μL of 50 mM acetic acid and adding 200 μL of 100 mM ammonium bicarbonate and 60 μL of acetonitrile in water. Trypsin solution was sprayed onto ITO glass slides using ImagePrep^TM^ (Bruker Daltonik GmbH, Bremen, Germany). The digestion was carried out at 37 °C for 18 h in a small humid box.

To maintain a humid atmosphere for a long-time trypsinization we have come with a solution how to prepare a humid chamber/humid box. During the optimization of our on-tissue trypsinization method, we have tried different setups (not shown), but the best setup was the one where we have used an old tip rack, which was filled with water and on top was placed Kimwipe tissue soaked in LC-MS water. The ITO glass slides with applied trypsin solution were placed on the soaked Kimwipe tissue and the humid box/tip rack was closed. For heating the box, we have used a magnetic stirrer with a hotplate. Our whole setup is presented in Figure S21. The other trick was with applying a trypsin solution. We have used the Bruker ImagePrep device, which is normally used for application of MALDI matrix for the tissue sections, but we have discussed that with our local Bruker representative and he send us a method for ImagePrep, which is used for application of trypsin solution, and then he showed us a trick how to put a small volume of trypsin solution (300 µL of trypsin solution vs. 5–10 mL used normally for MALDI matrix solution) by not using a normal glass container (with volume up to 10 mL) but using a pipette which tip was inserted as close as possible to the vibrating/spraying microgrid of the ImagePrep.

After the digest, the samples were dried under vacuum for 15 min. Then, MALDI matrix was sprayed onto ITO glass slides using ImagePrep standard programs. As MALDI matrix was used DHB in concentration of 30 mg/mL in 50% methanol and 1% trifluoroacetic acid (TFA). The samples were ready for MALDI MSI analysis after drying.

### 3.4. MALDI-TOF Mass Spectrometry Imaging

For mass spectrometry imaging was used the Bruker ultrafleXtreme MALDI-TOF/TOF mass spectrometer (Bruker Daltonik GmbH, Bremen, Germany). The MALDI MSI analysis and calibration of the instrument was performed according to optimized method described in Jarosova et al. [19]. The total sample set contained 48 cryosections on 24 ITO glass slides. There were 12 cryosections for each sample type: control lungs from non-infected pigs, lungs infected by APP—necrotic area (NA), marginal zone (MZ) and tracheobronchial lymph nodes (TBLN). The regions of measurement were set with 50 µm raster width. An external calibration was performed using a peptide calibration standard in an *m*/*z* range of 500–3500 Da. SCiLS Lab 2014b software (SCiLS–Bruker Daltonik GmbH, Bremen, Germany) was used for processing MSI spectra (baseline removal and normalization), generating MSI images and for MSI statistics. The parameters of MALDI MSI method were: reflector positive mode, *m*/*z* range of 500–3500 Da and the laser power was set to 85%. A total of 500 spectra were summed for each spot using a random walk raster pattern.

The LIFT cell of the instrument was used for MS/MS analysis of detected peptides [31,53]. To optimize parameters of LIFT method, the most intensive peak (1957.126 ± 0.100 Da) from MALDI MSI mean mass spectra of all measured sections (Figure S17) was selected. Then the parent and fragment ion mass spectra were measured on random positions of selected tissue sections. The laser power was set 5–10% above the threshold (the S/N ratio of the peak intensity was ≥10). Identification of detected peptides was achieved using the BioTools 3.2 (Bruker Daltonik GmbH, Bremen, Germany) and in-house MASCOT server (Matrix Science, Boston, MA, USA). The following parameters were used for MASCOT MS/MS Ion Search: NCBI protein database from 9th April 2019 was used, trypsin was used as the enzyme, zero or one missed cleavage was allowed, taxonomy was set to other *mammalia*, peptide mass tolerance was set to ±0.1 Da, fragment mass tolerance was set to ±0.5 Da with no variable modifications. The MASCOT score above 50 was chosen as significant. The results were displayed and analyzed in BioTools 3.2. The MASCOT results of Protegrin-4 precursor are presented in Figure S13, the LIFT mass spectrum with annotated fragments of Protegrin-4 precursor is presented in Figure S14, Protegrin-4 precursor protein sequence coverage covered by FPPPNFPGPR is presented in Figure S15 and LIFT spectrum analysis report is presented in Figure S16. The peptides of IL-1β and CD163 were not successfully identified based on LIFT fragment spectra, but they were identified by matching the measured *m*/*z* values with calculated values from in silico trypsinization of CD163 protein sequence and IL-1β protein sequence (obtained from UniProt database for *Sus scrofa*), and then they were confirmed by IHC. The in silico trypsinization was performed in SequenceEditor 3.2 (Bruker Daltonik GmbH, Bremen, Germany) using these parameters: trypsin was used as the enzyme, one or two missed cleavage was allowed and carbamidomethylation (C) was selected as optional modification.

### 3.5. Spectral Processing, Statistics and MSI Image Preparation

The MSI data were processed in SCiLS Lab 2014b software (SCiLS–Bruker Daltonik GmbH, Bremen, Germany) according to our previous study [32]: The preprocessing pipeline applied baseline removal using the iterative convolution algorithm with sigma = 20 and iterations = 20. Subsequently, the total ion current (TIC) of every spectrum was normalized to one. At the same time, the mean spectrum was calculated. The segmentation pipeline applied the spectral group resulting from the preprocessing pipeline to find peaks using orthogonal matching with sigma estimated from the mean spectrum. This process found a maximum of 50 peaks per spectrum and was applied to every 15th spectrum. The consensus threshold was 1%. Then, a peak alignment was applied to the resulting histogram peak list together with the mean spectrum. Finally, the denoising and segmentation pipeline was applied to the spectral group produced by the preprocessing pipeline, and the peak list was produced by the peak alignment. The denoising level was the same as that selected in the settings of the segmentation pipeline.

The Anderson-Darling normality test revealed non-normal distribution of MSI data, therefore a Kruskal–Wallis test was used. The semi-quantitation of peptides was based on their peak intensities only. All images/figures were finally prepared in GIMP 2.10.22 software.

### 3.6. Staining by H&E

H&E staining of cryosections after MALDI MSI was performed following the protocol of Kaya et al. [54]: MALDI matrix was washed away using 2 × 1 min submersions in 100% EtOH. Tissue was rehydrated in 70% EtOH, 50% EtOH, and Milli-Q water, 2 min each. The slide was placed in hematoxylin for 2 min and washed with water for 2 min. The slide was then counterstained in 0.2% eosin for 2 min and washed in water for 2 min. The section was finally washed and dehydrated in 50% EtOH, 70% EtOH, and 100% EtOH for 1 min each. Tissue was mounted with Entellan mounting medium. Some of the cryosections were washed away during the preparation, therefore other consecutive cryosections were used for staining. The pictures of stained sections were made by EVOS FL Auto Cell Imaging System (Thermo Fisher Scientific, Waltham, MA, USA) using scanning mode with 4× magnification.

### 3.7. Immunohistochemistry

The IHC analysis of IL-1β and CD163 was performed according to Jarosova et al. [19]: The endogenous peroxidase was blocked with Dual Endogenous Enzyme-Blocking Reagent (DAKO, Glostrup, Denmark) for 10 min. Slides were washed and the Protein Block (DAKO, Glostrup Denmark) was applied for 5 min. Then, primary antibodies of cytokines: anti-CD163 (clone 2A10/11, IgG1 subclass, dilution 1:800, Serotec, Oxford, UK) and anti-porcine IL-1beta (IgG subclass, dilution 1:600, Antibodies-online, Aachen, Germany) were applied and the slides were incubated for 60 min at 37 °C in a humid chamber. Then, the slides were washed and EnVision reagent HRP anti-Mouse or anti-Rabbit (DAKO, Glostrup, Denmark) was added. The slides were incubated for 30 min at 37 °C in a humid chamber. As a negative control, immunohistochemistry was performed using secondary antibodies without the primary antibody. The slides were washed and stained with DAB + Liquid (DAKO, Glostrup Denmark) for approx. 30 sec. Then, the slides were washed with distilled water, lightly counterstained with Mayer hematoxylin (PENTA s.r.o., Praha, Czech Republic) and mounted in glycerol-gelatin.

The histological slides were evaluated by using the light microscope Olympus BH-2 (Olympus, Japan), two magnifications 100× and 400× were used. Images of sections were taken with a VH-Z500R high resolution zoom lens (Keyence, Itasca, IL, USA) mounted on a Keyence VHX-5000 digital microscope (Keyence, Itasca, IL, USA), magnification 300× was used. The original IHC images were modified in GIMP 2.10.22 by changing the contrast, brightness and RGB values. The original and modified IHC images are presented in Figures S1–S6 for IL-1β and in Figures S7–S12 for CD163.

## 4. Conclusions

Using MALDI-TOF mass spectrometry imaging, we were able to map spatial distribution of CD163, IL-1β and protegrin-4 precursor in cryosections of porcine lungs and tracheobronchial lymph nodes, based on their tryptic fragments after on-tissue trypsinization. The presence of CD163 and IL-1β was confirmed also by IHC; moreover, the MALDI MSI added more information for positions on sections where IHC antibody was not bound properly. For protegrin-4 precursor, there is no suitable IHC antibody, but we were able to identify it directly on-tissue using MALDI-LIFT-TOF/TOF and MASCOT. CD163, IL-1β and protegrin-4 precursor were significantly (*p* < 0.001; Kruskal–Wallis test) more expressed in porcine lungs infected by APP—necrotic area (NA) than in other samples, like control lungs or marginal zone (MZ) and tracheobronchial lymph nodes (TBLN) from APP-infected pigs. From the results we can conclude that MALDI-TOF MSI is suitable for analyzing infectious markers in porcine lung tissue and it is a complementary method to other imaging methods such as IHC. It can be used for detection of markers for which the IHC antibody does not exist, as it is in a case of protegrin-4 precursor. The applicability of MALDI-TOF MSI and other MSI methods therefore surpasses IHC from this point of view. Moreover, the co-detection of higher expression of protegrin-4 precursor with the higher expression of IL-1β can give us more information about APP infection of porcine lungs and therefore should be studied more. 

## Figures and Tables

**Table 1:** Kruskal–Wallis tests for CD163, IL-1β and protegrin-4 precursor tryptic fragments/peptides. The statistical significance of differences between lungs control, lungs infected by APP—NA, MZ and TBLN was tested.

	*m*/*z* [Da]	p _Lungs Control vs NA	p_Lungs Control vs TBLN	p_Lungs Control vs MZ	p_NA vs TBLN	p_NA vs MZ	p_MZ vs TBLN
**CD163**	975.478	<0.001	<0.001	<0.001	<0.001	<0.001	<0.001
**Protegrin-4 Precursor**	1126.216	<0.001	<0.001	<0.001	<0.001	<0.001	<0.001
**IL-1β**	3119.621	<0.001	<0.001	<0.001	<0.001	<0.001	<0.001

## Supplementary Materials

The following are available online. Figure S1: IHC image of IL-1β in cryosection of control/healthy lung before and after modification in GIMP 2.10.22. The length of scale bar is 1 mm. The positive IHC signal has purple color in modified image. Figure S2: IHC image of IL-1β in cryosection of first necrotic area (NA) from lungs obtained from pigs infected with APP, before and after modification in GIMP 2.10.22. The length of scale bar is 1 mm. The positive IHC signal has purple color in modified image. Figure S3: IHC image of IL-1β in cryosection of second necrotic area (NA) from lungs obtained from pigs infected with APP, before and after modification in GIMP 2.10.22. The length of scale bar is 1 mm. The positive IHC signal has light purple color in modified image. Figure S4: IHC image of IL-1β in cryosection of third necrotic area (NA) from lungs obtained from pigs infected with APP, before and after modification in GIMP 2.10.22. The length of scale bar is 1 mm. The positive IHC signal has purple color in modified image. Figure S5: IHC image of IL-1β in cryosection of tracheobronchial lymph node (TBLN) obtained from pigs infected with APP, before and after modification in GIMP 2.10.22. The length of scale bar is 1 mm. The positive IHC signal has purple color in modified image. Figure S6: IHC image of IL-1β in cryosection of marginal zone (MZ) from lungs obtained from pigs infected with APP, before and after modification in GIMP 2.10.22. The length of scale bar is 1 mm. The positive IHC signal has purple color in modified image. Figure S7: IHC image of IL-1β in cryosection of control/healthy lung before and after modification in GIMP 2.10.22. The length of scale bar is 1 mm. The positive IHC signal has brown color in modified image. Figure S8: IHC image of IL-1β in cryosection of first necrotic area (NA) from lungs obtained from pigs infected with APP, before and after modification in GIMP 2.10.22. The length of scale bar is 1 mm. The positive IHC signal has brown color in modified image. Figure S9: IHC image of IL-1β in cryosection of second necrotic area (NA) from lungs obtained from pigs infected with APP, before and after modification in GIMP 2.10.22. The length of scale bar is 1 mm. The positive IHC signal has brown/grey color in modified image. Figure S10: IHC image of IL-1β in cryosection of third necrotic area (NA) from lungs obtained from pigs infected with APP, before and after modification in GIMP 2.10.22. The length of scale bar is 1 mm. The positive IHC signal has brown color in modified image. Figure S11: IHC image of IL-1β in cryosection of tracheobronchial lymph node (TBLN) obtained from pigs infected with APP, before and after modification in GIMP 2.10.22. The length of scale bar is 1 mm. The positive IHC signal has brown color in modified image. Figure S12: IHC image of IL-1β in cryosection of marginal zone (MZ) from lungs obtained from pigs infected with APP, before and after modification in GIMP 2.10.22. The length of scale bar is 1 mm. The positive IHC signal has brown color in modified image. Figure S13: Results of identification of protegrin-4 precursor from MASCOT. Figure S14: LIFT mass spectrum with annotated fragments of protegrin-4 precursor peptide FPPPNFPGPR. BioTools 3.2 was used for annotation. Figure S15: Protegrin-4 precursor protein sequence coverage covered by FPPPNFPGPR. The sequence coverage is low, but the identified peptide is unique for this protein family. Figure S16: The LIFT spectrum analysis report for protegrin-4 precursor peptide FPPPNFPGPR. BioTools 3.2 was used for analysis. Figure S17: The MALDI MSI mean mass spectrum created from all measured tissue sections. SCiLS Lab 2014b was used for processing the spectra applying baseline removal and normalization. Figure S18: Zoomed MALDI MSI mean mass spectrum created from all measured tissue sections showing the peak of IL-1β peptide NLYLSCVLKDDKPTLQLESVDPKNYPK. SCiLS Lab 2014b was used for processing the spectra applying baseline removal and normalization. Figure S19: Zoomed MALDI MSI mean mass spectrum created from all measured tissue sections showing the peak of protegrin-4 precursor peptide FPPPNFPGPR. SCiLS Lab 2014b was used for processing the spectra applying baseline removal and normalization. Figure S20: Zoomed MALDI MSI mean mass spectrum created from all measured tissue sections showing the peak of CD163 peptide TSYQVYSK. SCiLS Lab 2014b was used for processing the spectra applying baseline removal and normalization. Figure S21: The preparation of humid box used for on-tissue trypsinization. (A) A magnetic stirrer with hotplate used for heating the humid box. (B) A humid box prepared from used tip rack, which was filled with water and on top was placed Kimwipe tissue soaked in LC-MS water. The ITO glass slides with applied trypsin solution were placed on the soaked Kimwipe tissue and the humid box/tip rack was closed. (C) A final set up used for 18h trypsinization.
