# Supplementary material for: MALDI MSI Reveals the Spatial Distribution of Protein Markers in Tracheobronchial Lymph Nodes and Lung of Pigs after Respiratory Infection
Source: Molecules. 2020 Dec 3;25(23):5723. doi: 10.3390/molecules25235723 (PMC7730995; doi:10.3390/molecules25235723)
Supplement: Supplementary file 1 [file molecules-25-05723-s001.pdf]

MALDI MSI Reveals the Spatial Distribution of Protein Markers in Tracheobronchial Lymph Nodes and Lung of Pigs after Respiratory Bacterial Infection

Tomas Do [1,†], Roman Guran [1,2,†], Rea Jarosova [3], Petra Ondrackova [4], Zbysek Sladek [3], Martin Faldyna [4], Vojtech Adam [1,2,5] and Ondrej Zitka [1,2,5*]

[1] Department of Chemistry and Biochemistry, Faculty of AgriSciences, Mendel University in Brno, Brno, Czech Republic; xdo1@mendelu.cz (T.D.), roman.guran@mendelu.cz (R.G.), vojtech.adam@mendelu.cz (V.A.), ondrej.zitka@mendelu.cz (O.Z.)
[2] Central European Institute of Technology, Brno University of Technology, Brno, Czech Republic
[3] Department of Morphology, Physiology and Animal Genetics, Faculty of AgriSciences, Mendel University in Brno, Brno, Czech Republic; rea.jarosova@mendelu.cz (R.J.), zbysek.sladek@mendelu.cz (Z.S.)
[4] Department of Immunology, Veterinary Research Institute, Brno, Czech Republic; ondrackovap@vri.cz (P.O.), faldyna@vri.cz (M.F.)
[5] Central European Institute of Technology, Mendel University in Brno, Brno, Czech Republic
* Correspondence: ondrej.zitka@mendelu.cz; Tel.: +420 545 133 285, +420 545 133 350 (O.Z.)
† These authors contributed equally to this work.

Original and modified IHC images of IL-1β including cryosections from control/healthy lung and necrotic area (NA), marginal zone (MZ) and tracheobronchial lymph node (TBLN) from pigs infected by *Actinobacillus pleuropneumoniae* (APP)

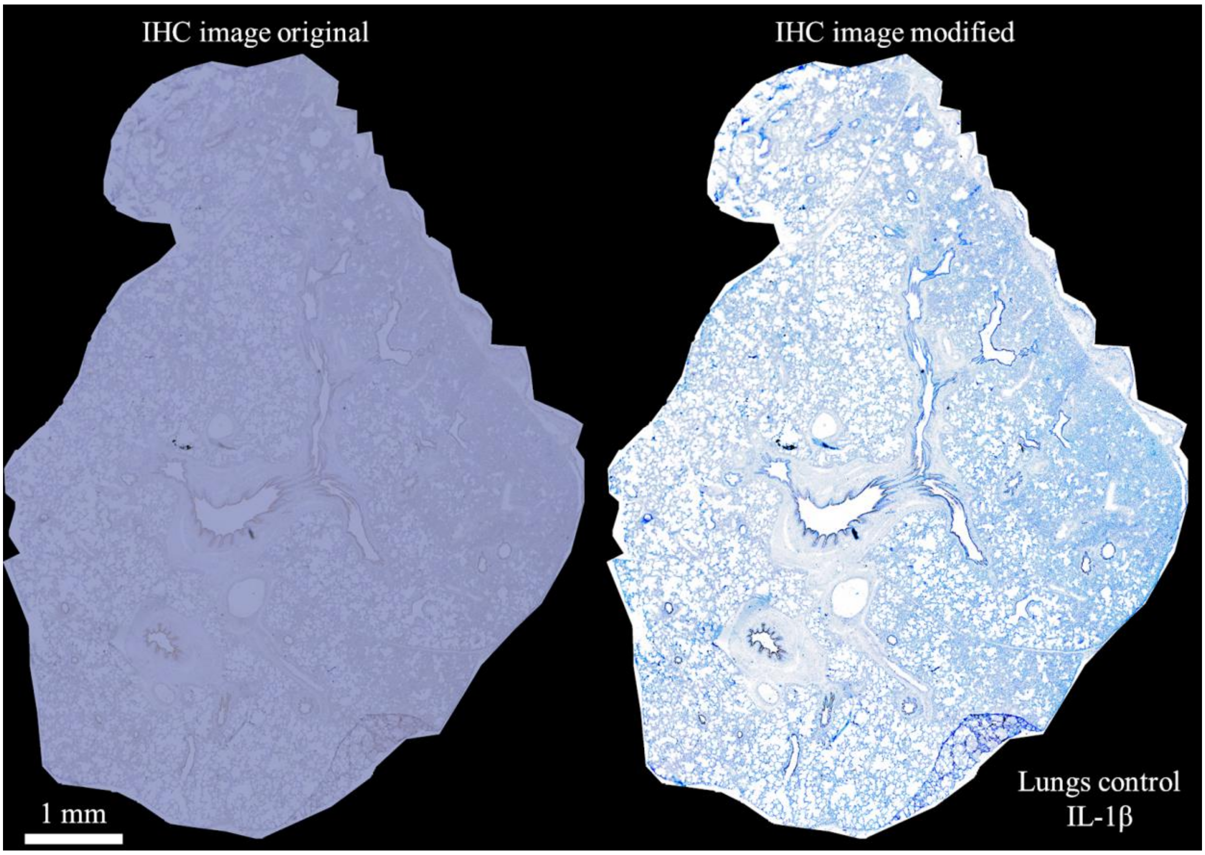

Figure S1. IHC image of IL-1β in cryosection of control/healthy lung before and after modification in GIMP 2.10.22. The length of scale bar is 1 mm. The positive IHC signal has purple color in modified image.

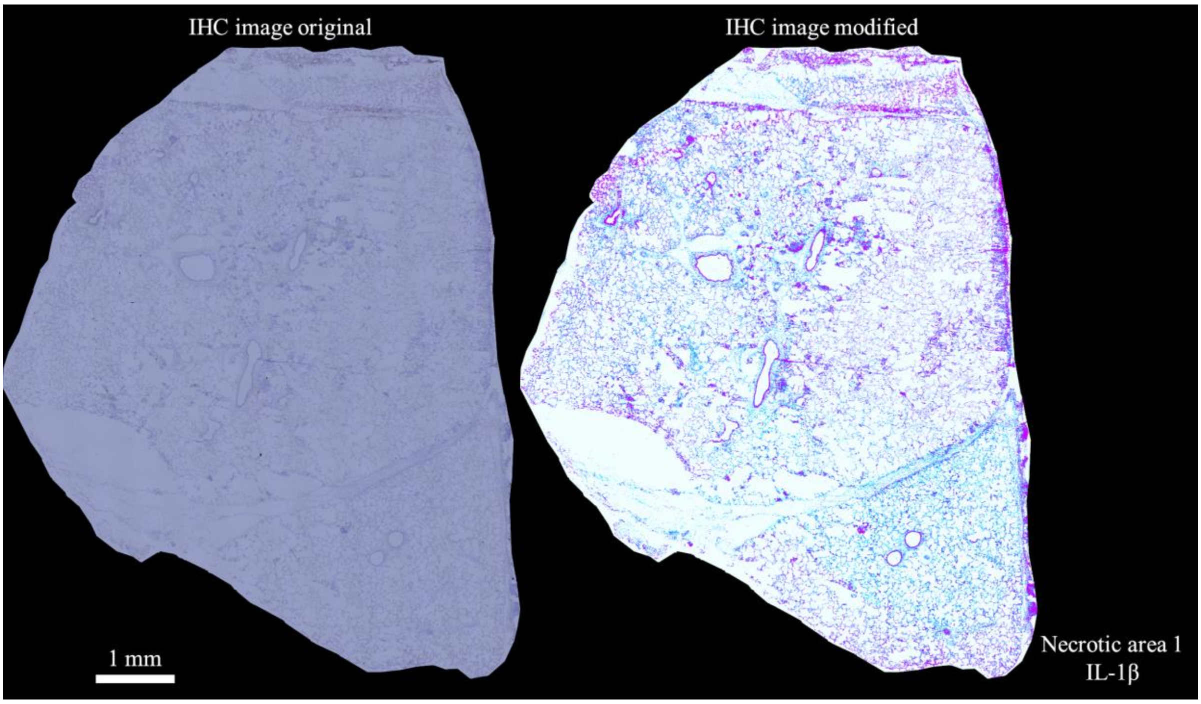

Figure S2. IHC image of IL-1β in cryosection of first necrotic area (NA) from lungs obtained from pigs infected with APP, before and after modification in GIMP 2.10.22. The length of scale bar is 1 mm. The positive IHC signal has purple color in modified image.

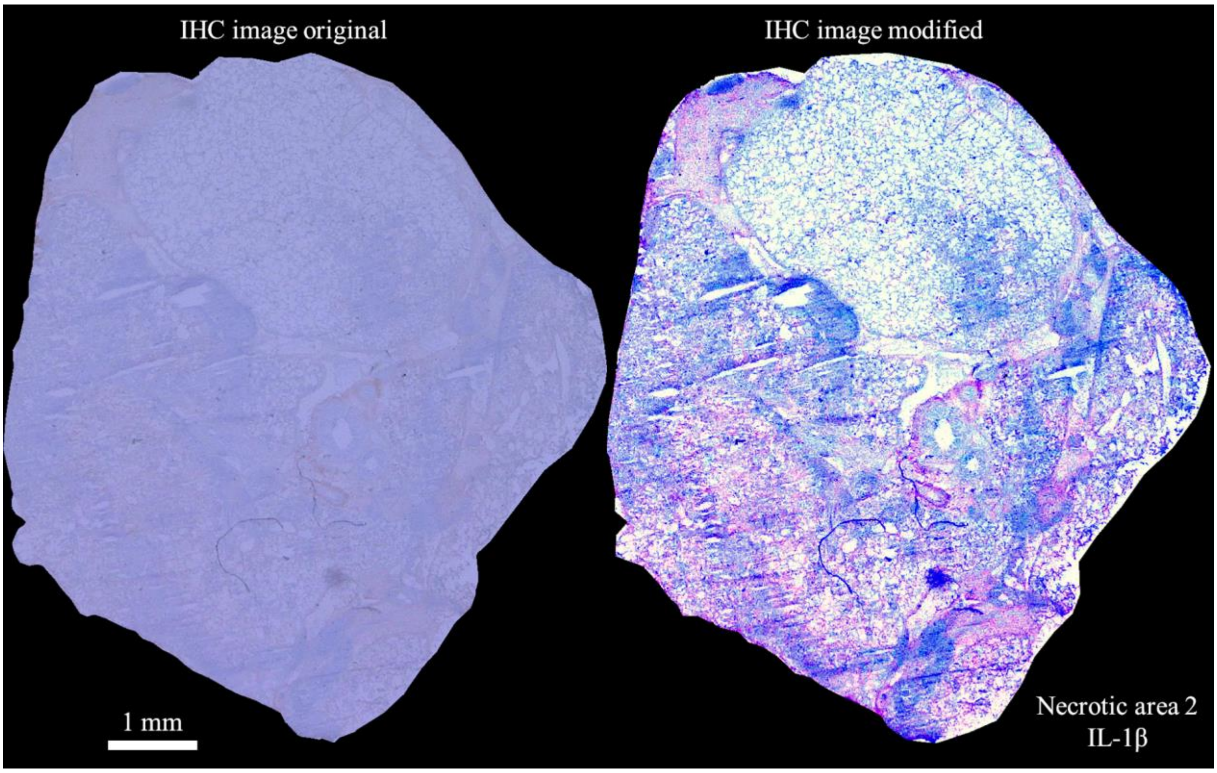

Figure S3. IHC image of IL-1β in cryosection of second necrotic area (NA) from lungs obtained from pigs infected with APP, before and after modification in GIMP 2.10.22. The length of scale bar is 1 mm. The positive IHC signal has light purple color in modified image.

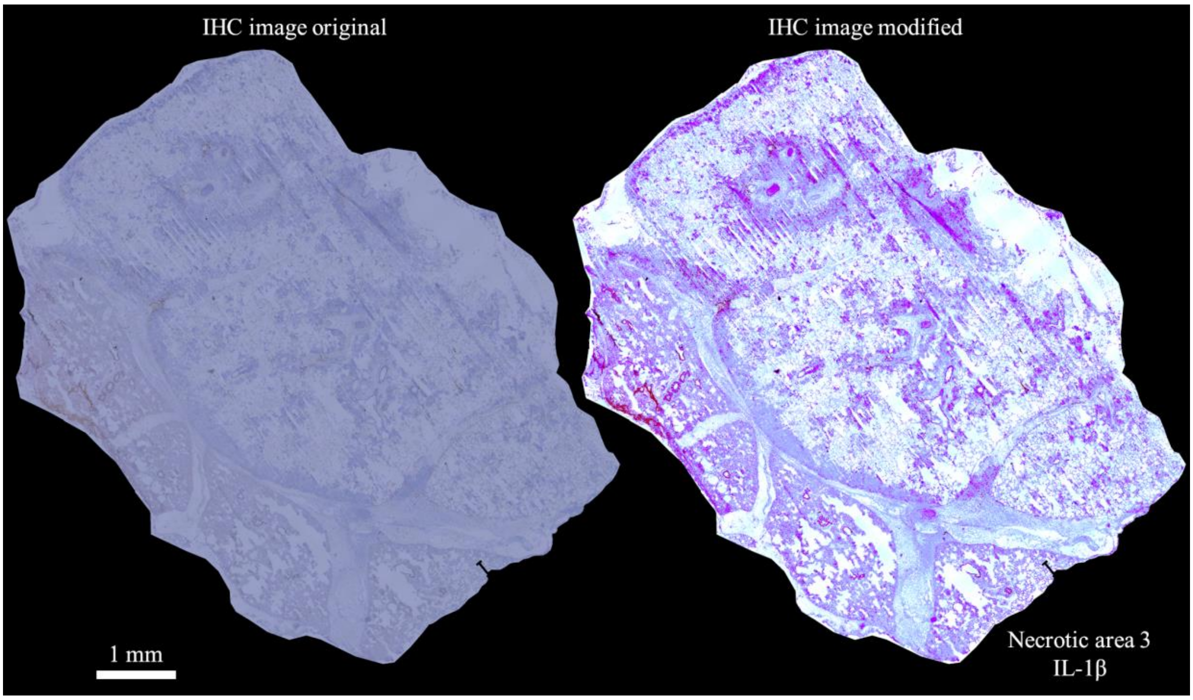

Figure S4. IHC image of IL-1β in cryosection of third necrotic area (NA) from lungs obtained from pigs infected with APP, before and after modification in GIMP 2.10.22. The length of scale bar is 1 mm. The positive IHC signal has purple color in modified image.

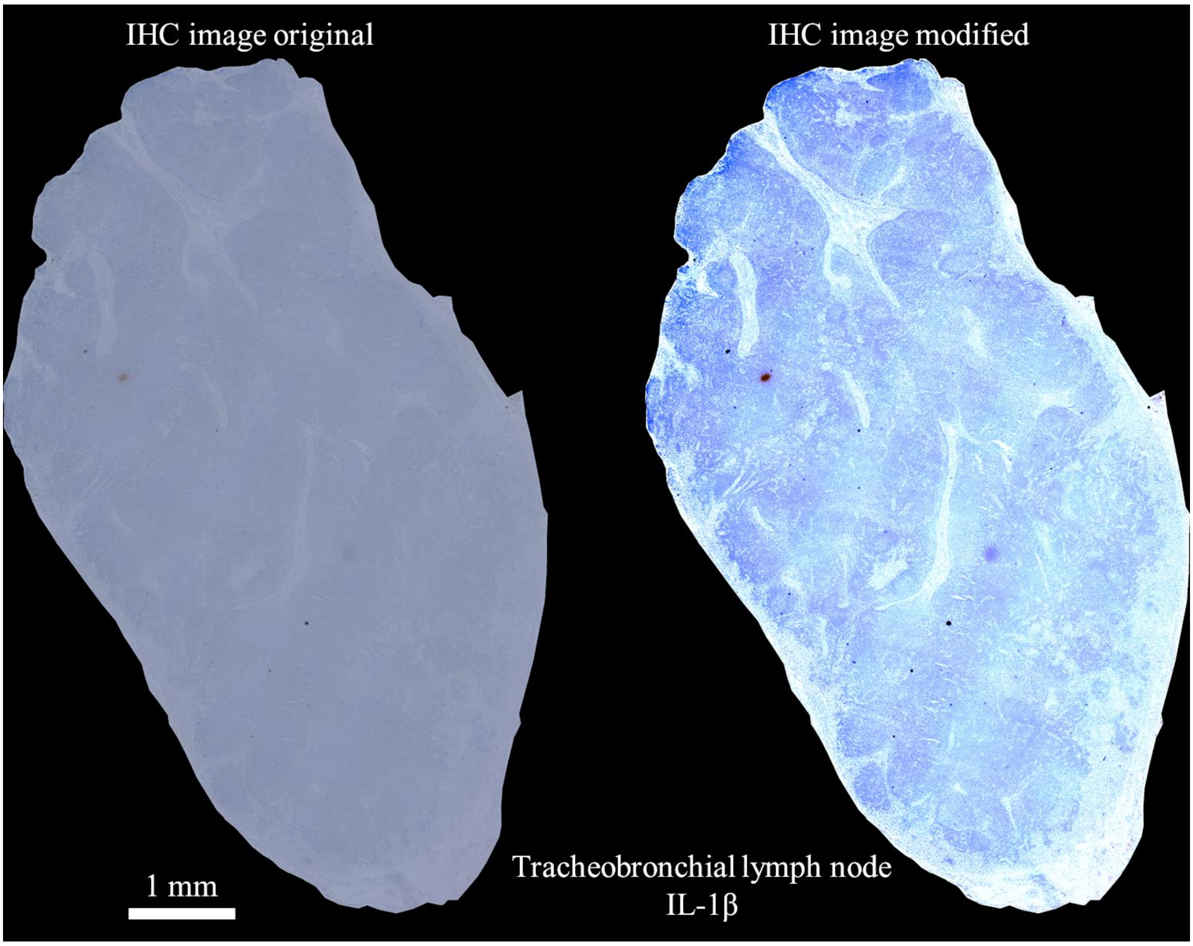

Figure S5. IHC image of IL-1β in cryosection of tracheobronchial lymph node (TBLN) obtained from pigs infected with APP, before and after modification in GIMP 2.10.22. The length of scale bar is 1 mm. The positive IHC signal has purple color in modified image.

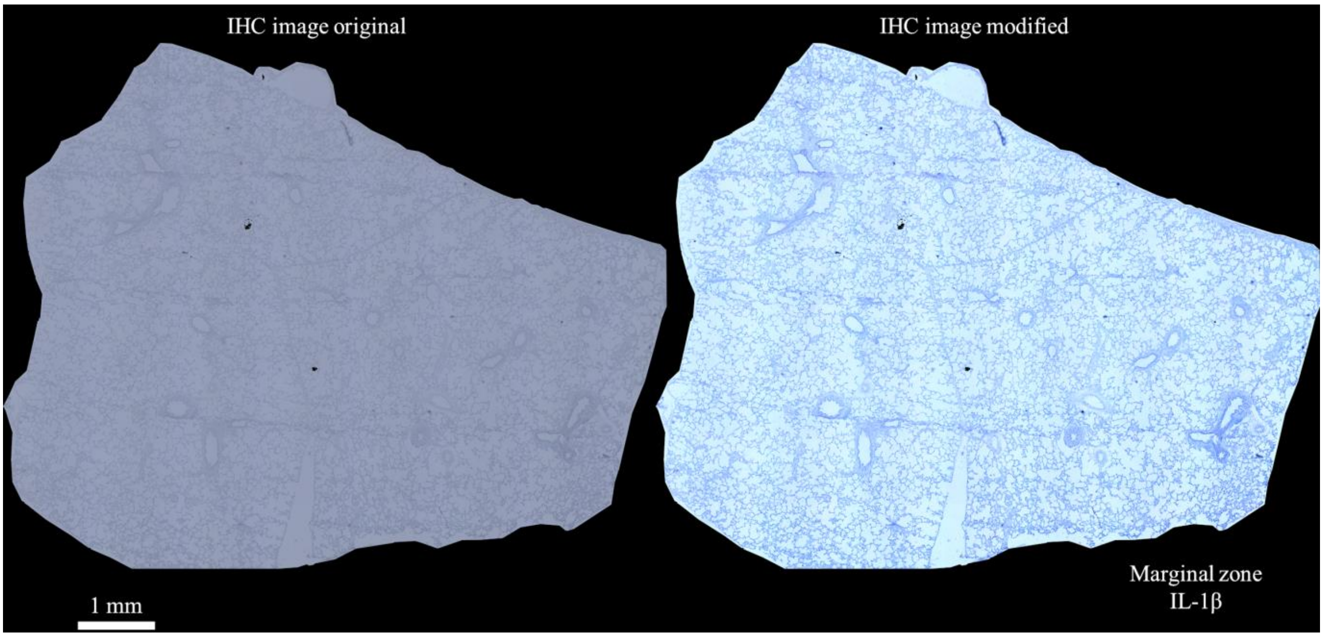

Figure S6. IHC image of IL-1β in cryosection of marginal zone (MZ) from lungs obtained from pigs infected with APP, before and after modification in GIMP 2.10.22. The length of scale bar is 1 mm. The positive IHC signal has purple color in modified image.

Original and modified IHC images of CD163 including cryosections from control/healthy lung and necrotic area (NA), marginal zone (MZ) and tracheobronchial lymph node (TBLN) from pigs infected by *Actinobacillus pleuropneumoniae* (APP)

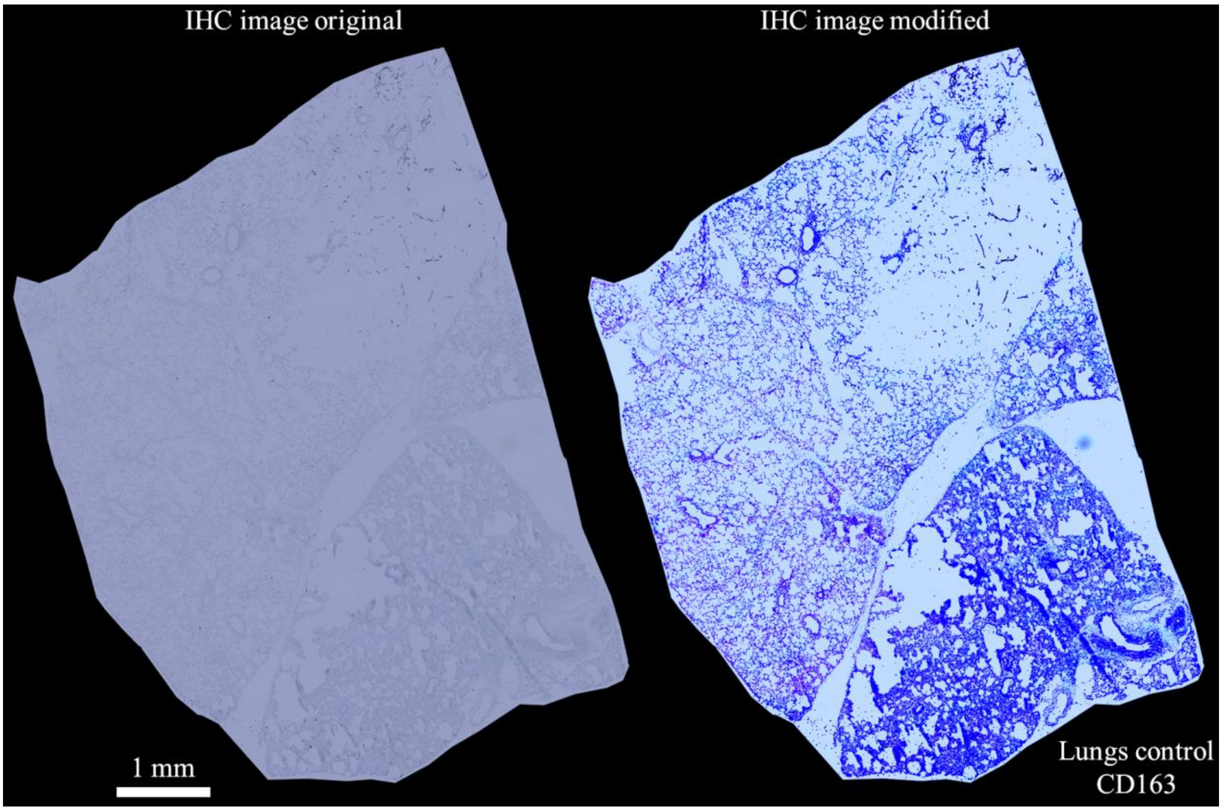

Figure S7. IHC image of IL-1β in cryosection of control/healthy lung before and after modification in GIMP 2.10.22. The length of scale bar is 1 mm. The positive IHC signal has brown color in modified image.

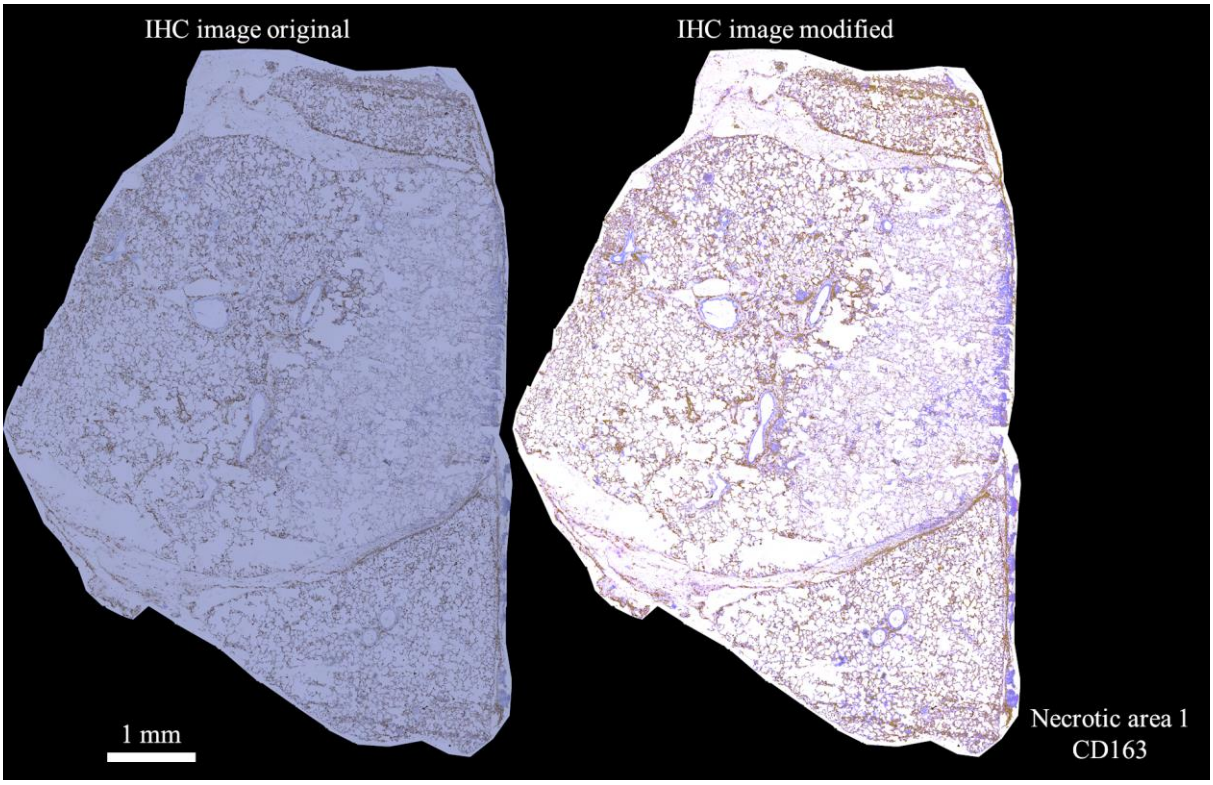

Figure S8. IHC image of IL-1β in cryosection of first necrotic area (NA) from lungs obtained from pigs infected with APP, before and after modification in GIMP 2.10.22. The length of scale bar is 1 mm. The positive IHC signal has brown color in modified image.

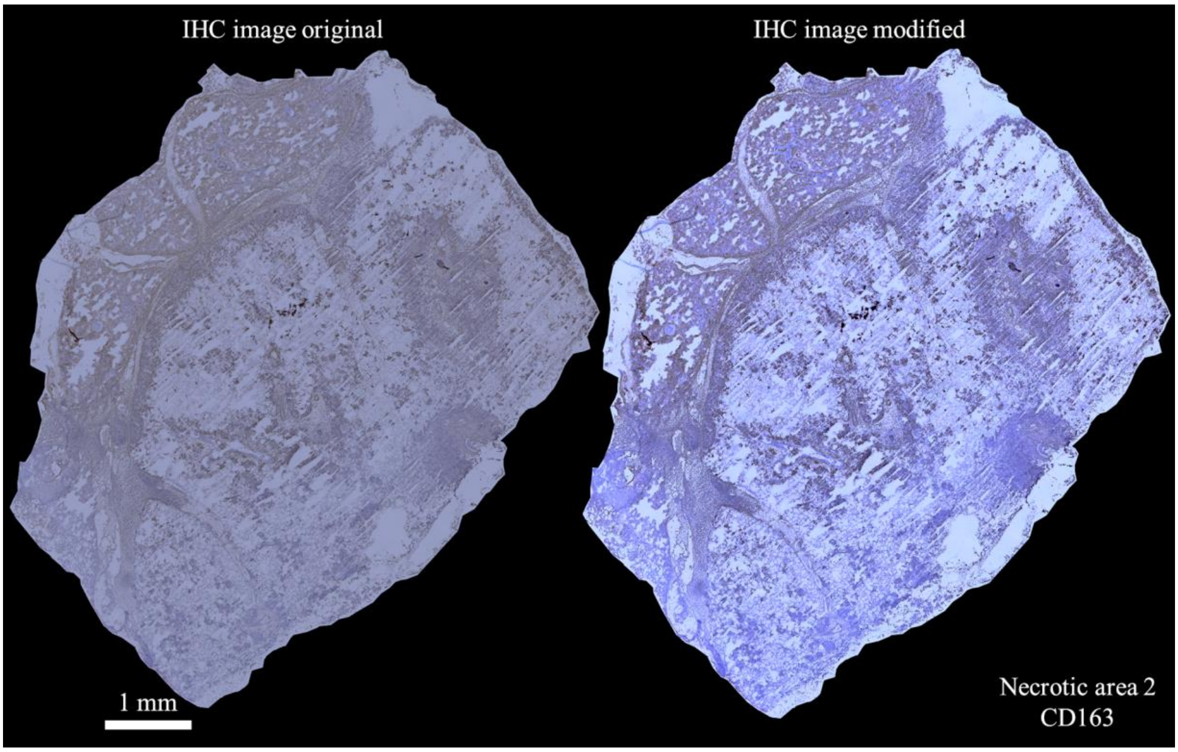

Figure S9. IHC image of IL-1β in cryosection of second necrotic area (NA) from lungs obtained from pigs infected with APP, before and after modification in GIMP 2.10.22. The length of scale bar is 1 mm. The positive IHC signal has brown/grey color in modified image.

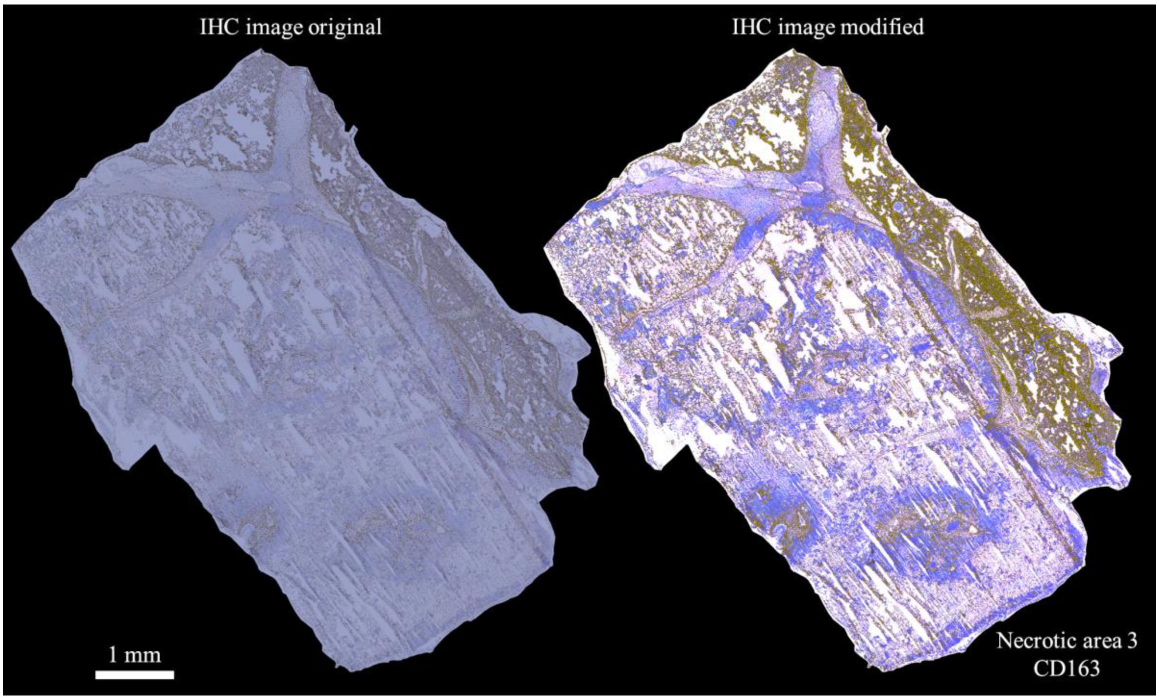

Figure S10. IHC image of IL-1β in cryosection of third necrotic area (NA) from lungs obtained from pigs infected with APP, before and after modification in GIMP 2.10.22. The length of scale bar is 1 mm. The positive IHC signal has brown color in modified image.

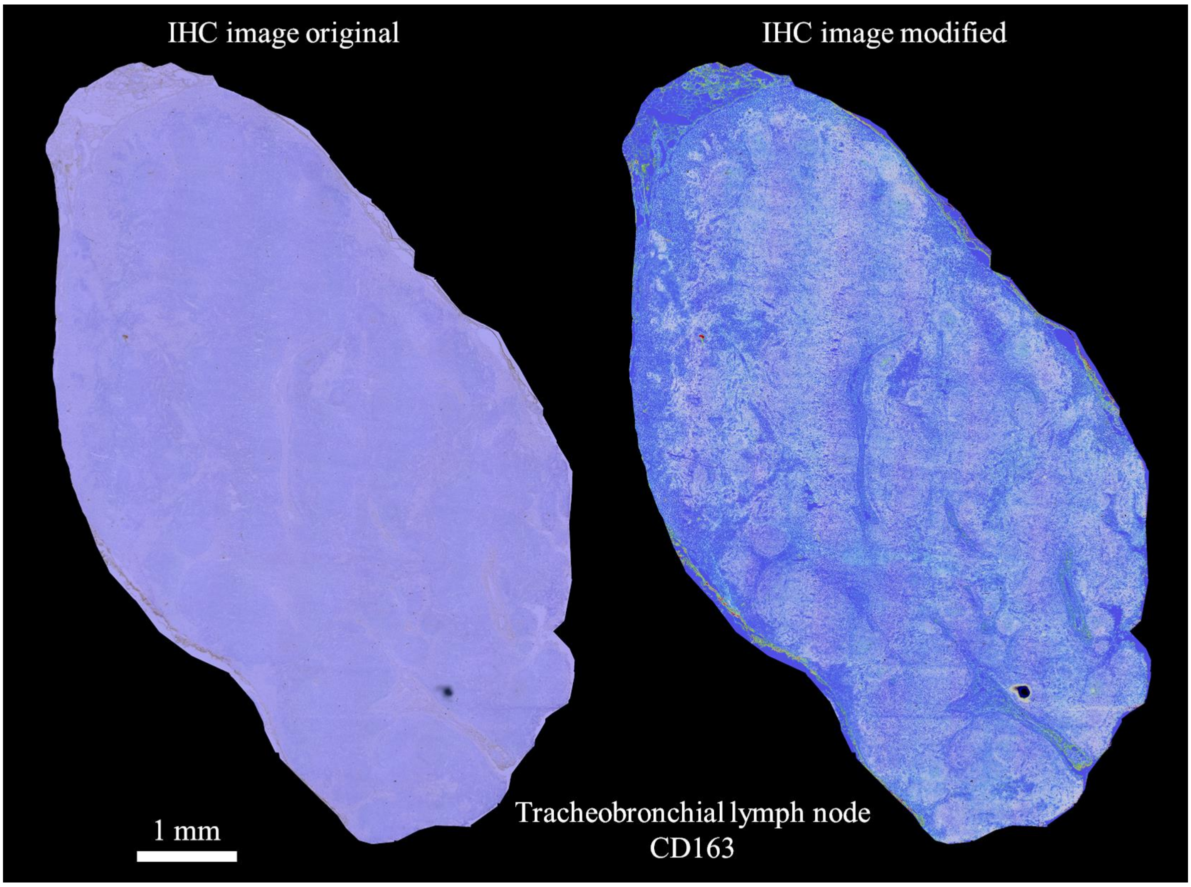

Figure S11. IHC image of IL-1β in cryosection of tracheobronchial lymph node (TBLN) obtained from pigs infected with APP, before and after modification in GIMP 2.10.22. The length of scale bar is 1 mm. The positive IHC signal has brown color in modified image.

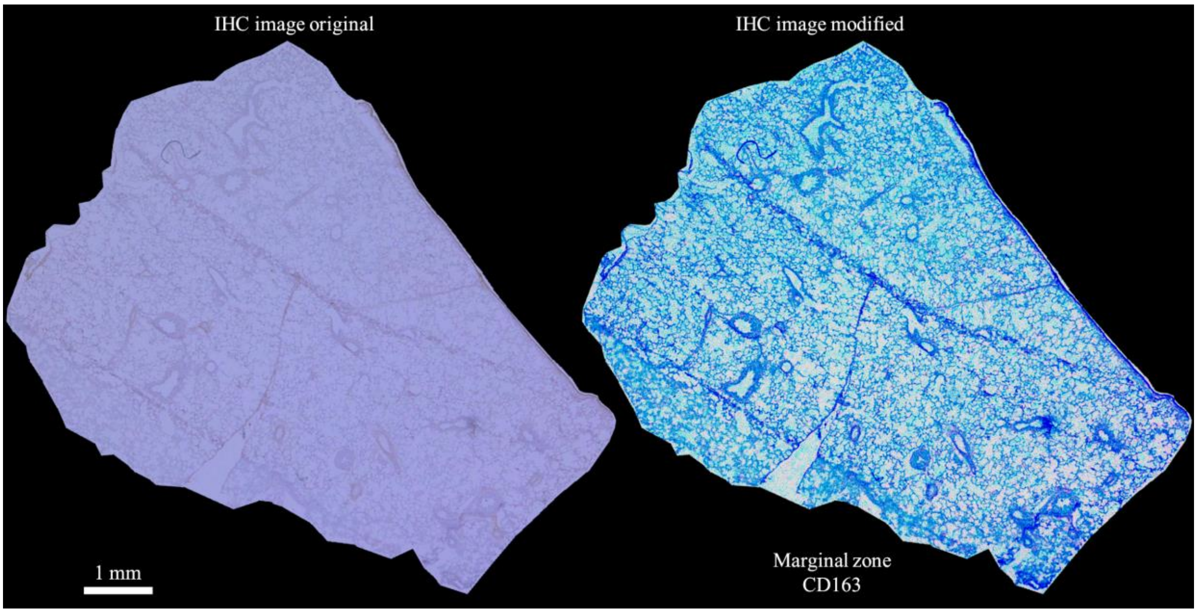

Figure S12. IHC image of IL-1β in cryosection of marginal zone (MZ) from lungs obtained from pigs infected with APP, before and after modification in GIMP 2.10.22. The length of scale bar is 1 mm. The positive IHC signal has brown color in modified image.

Identification of protegrin-4 precursor by MALDI-LIFT-TOF/TOF and MASCOT

Mascot Search Results

User	:
Email	:
Search title	:
MS data file	: DATA.TXT
Database	: NCBIprot 20190409 (198058129 sequences; 72054219131 residues)
Taxonomy	: Other mammalia (2621646 sequences)
Timestamp	: 10 Dec 2019 at 15:13:04 GMT
Warning	: There is an error in a configuration file: Error in the configuration file ..\config\mascot.dat - database UniProt_proteome_
Warning	: 2 sequences ignored because length greater than maximum configured
Protein hits	: NP_999028.1 protegrin-4 precursor [Sus scrofa]
	XP_022368645.1 dihydrolipoyllysine-residue acetyltransferase component of pyruvate dehydrogenase complex, mitochondrial [Enh
	XP_006873889.1 PREDICTED: protein ITFG3 [Chrysochloris asiatica]
	XP_012782233.1 PREDICTED: centromere protein J [Ochotona princeps]
	XP_023392235.1 ubiquitin-associated domain-containing protein 1 [Pteropus vampyrus]
	XP_024436573.1 LOW QUALITY PROTEIN: sushi domain-containing protein 3 [Desmodus rotundus]
	EPY83234.1 hypothetical protein CB1_000589041 [Camelus ferus]
	XP_007940149.1 PREDICTED: homeobox protein Hox-B9 [Orycteropus afer afer]
	XP_019837838.1 PREDICTED: sorting nexin-11 isoform X1 [Bos indicus]
	XP_008685282.1 PREDICTED: xin actin-binding repeat-containing protein 2 isoform X1 [Ursus maritimus]

Mascot Score Histogram

Ions score is -10*Log(P), where P is the probability that the observed match is a random event.
Individual ions scores > 42 indicate peptides with significant homology.
Individual ions scores > 47 indicate identity or extensive homology (p<0.05).
Protein scores are derived from ions scores as a non-probabilistic basis for ranking protein hits.

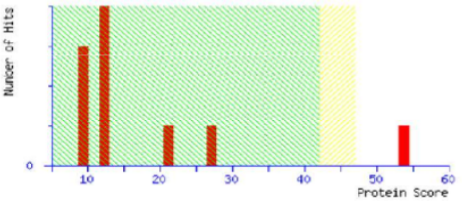

Peptide Summary Report

Format As: Peptide Summary — Help

Significance threshold p< 0.05 — Max. number of hits 20

Standard scoring ◉ MudPIT scoring ○ — Ions score or expect cut-off 0 — Show sub-sets 0

Show pop-ups ◉ Suppress pop-ups ○ — Sort unassigned Decreasing Score — Require bold red ☐

Preferred taxonomy All entries

Overview Table

Click on column header to jump to entry in results list.
Move mouse over any indicator to highlight identical peptides.
Click on an indicator to see details of individual match.
Use check boxes to select sub-set of queries for new search.

Mouse over: -Query- -Accession- -Sequence-

Hit:	1	2	3	4	5	6	7	8	9	10
☑ 1125.4860 (1+)	●	●	●	●	●	●	●	●	●	●

Select All — Select None — Search Selected — ☐ **Error tolerant** — Archive Report

1. NP_999028.1 **Mass:** 25839 **Score:** 54 **Matches:** 1(1) **Sequences:** 1(1)
   protegrin-4 precursor [Sus scrofa]
   ☐ Check to include this hit in error tolerant search or archive report

Query	Observed	Mr(expt)	Mr(calc)	Delta	Miss	Score	Expect	Rank	Unique	Peptide
1	1125.4860	1124.4787	1124.5767	-0.0980	0	55	0.0084	1	U	R.FPPPNFPGPR.F

Proteins matching the same set of peptides:
AAB33924.1 **Mass:** 8675 **Score:** 53 **Matches:** 1(1) **Sequences:** 1(1)
prophenin-1, PF-1=antimicrobial peptide {decamer repeats} [swine, leukocytes, Peptide, 79 aa]
P51524.1 **Mass:** 23941 **Score:** 53 **Matches:** 1(1) **Sequences:** 1(1)
RecName: Full=Prophenin and tritrpticin precursor; AltName: Full=C6; Contains: RecName: Full=Prophenin-1; Short=PF-1; Contains: RecName:
XP_020924182.1 **Mass:** 29255 **Score:** 51 **Matches:** 1(1) **Sequences:** 1(1)
protegrin-4 isoform X1 [Sus scrofa]

---

2. XP_022368645.1 **Mass:** 68935 **Score:** 27 **Matches:** 1(0) **Sequences:** 1(0)
   dihydrolipoyllysine-residue acetyltransferase component of pyruvate dehydrogenase complex, mitochondrial [Enhydra lutris kenyoni]
   ☐ Check to include this hit in error tolerant search or archive report

Query	Observed	Mr(expt)	Mr(calc)	Delta	Miss	Score	Expect	Rank	Unique	Peptide
1	1125.4860	1124.4787	1124.5284	-0.0497	0	27	6.3	2	U	R.EDPGAPCLAPR.A

Figure S13. Results of identification of protegrin-4 precursor from MASCOT.

Spectrum Analysis Report

Date: 11/19/2020 Time: 07:16

FileName: D:\MALDI-data\Roman Guran\2019\20190131_Prase_IGA_LIFT\20190206_IGA_Prase_plice_HCCA_fragments_of_1125.4860\0_D9\1\1125.4860.LIFT\1SRef\pdata\1\1r

Sequence Name:		Formula:		Parentmass:	1125.486
Mass Error:	-0.099	MH+ (mono):	1125.585	MH+ (avg):	1126.289
Threshold (a.i.):	300.000	Tolerance (Da):	0.500	Number of Peaks:	68
Above Threshold:	60	Assigned Peaks:	24	Not assigned Peaks:	44

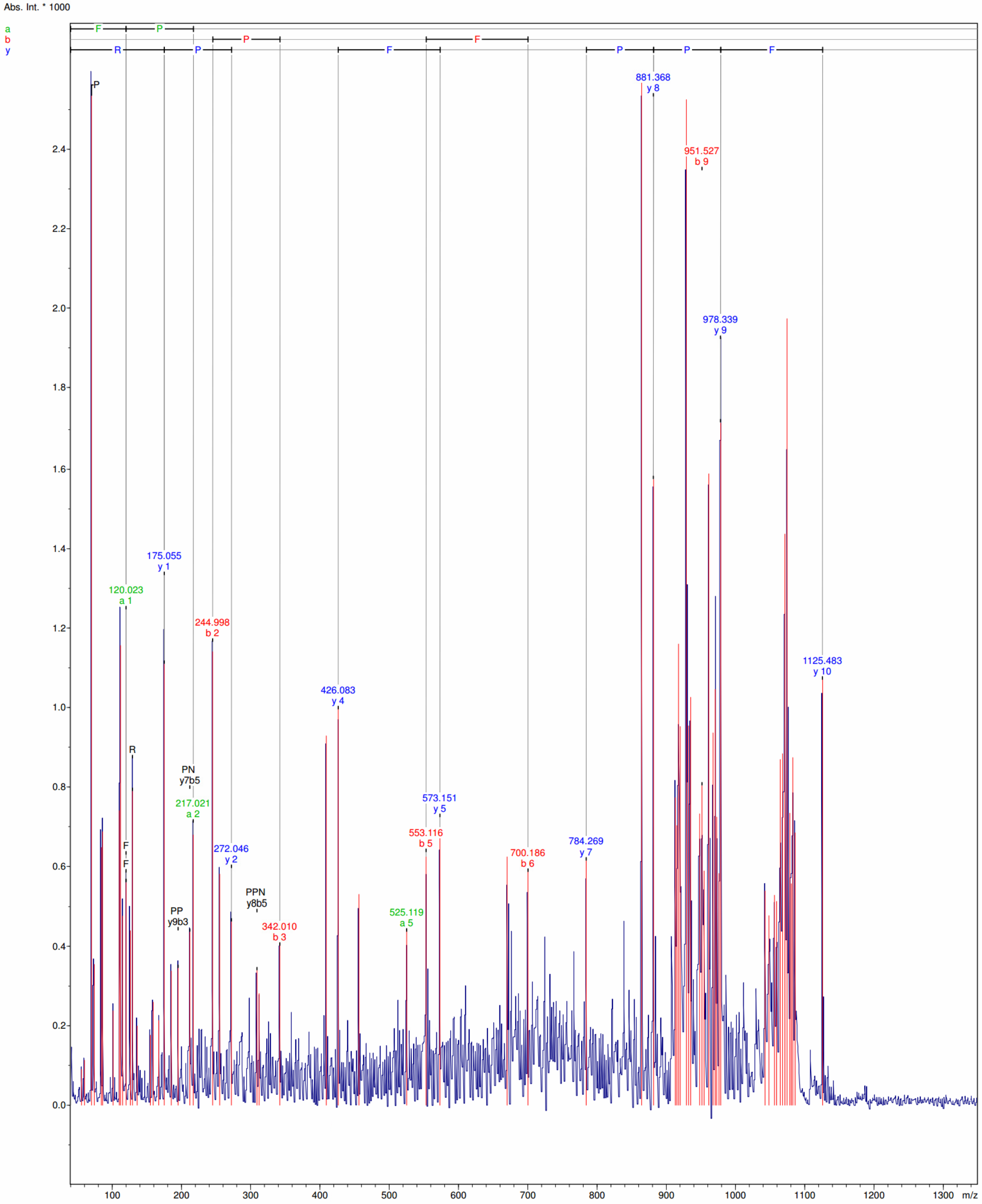

Copyright © 1999-2008 Bruker Daltonik GmbH

Figure S14. LIFT mass spectrum with annotated fragments of protegrin-4 precursor peptide FPPPNFPGPR. BioTools 3.2 was used for annotation.

Protein sequence coverage: 4%

Matched peptides shown in ***bold red***.

```
  1 METQRASLCL GRWSLWLLLL ALVVPSASAQ ALSYREAVLR AVDRLNEQSS
 51 EANLYRLLEL DQPPKADEDP GTPKPVSFTV KETVCPRPTR RPPELCDFKE
101 NGRVKQCVGT VTLDQIKDPL DITCNEGVRR FPWWWPFLRR PRLRRQAFPP
151 PNVPGPRFPP PNVPGPRFPP PNFPGPRFPP PNFPGPRFPP PNFPGPPFPP
201 PIFPGPWFPP PPPFRPPPFG PPRFPGRR
```

Figure S15. Protegrin-4 precursor protein sequence coverage covered by FPPPNFPGPR. The sequence coverage is low, but the identified peptide is unique for this protein family.

Spectrum Analysis Report

Date: 11/19/2020 Time: 07:16

FileName: D:\MALDI-data\Roman Guran\2019\20190131_Prase_IGA_LIFT\20190206_IGA_Prase_plice_HCCA_fragments_of_1125.4860\0_D9\1\1125.4860.LIFT\1SRef\pdata\1\1r

Display Parameter:

Parentmass:	1125.486	Mass Error:	-0.099	MH+ (mono):	1125.585
MH+ (avg):	1126.289	Threshold (a.i.):	300.000	Tolerance (Da):	0.500
Number of Peaks:	68	Above Threshold:	60	Assigned Peaks:	24
Not assigned Peaks:	44				

Peaklist:

Peak	Mass	Intensity	Peak	Mass	Intensity	Peak	Mass	Intensity	Peak	Mass	Intensity
1	55.627	90.331	2	59.983	113.497	3	70.017	2535.537	4	74.079	354.834
5	84.047	647.695	6	86.059	687.454	7	101.044	237.881	8	110.042	740.743
9	112.048	1156.413	10	115.060	476.701	11	120.023	559.953	12	126.051	439.586
13	129.072	789.606	14	136.028	199.834	15	155.030	176.587	16	159.045	260.217
17	167.011	213.788	18	175.055	1109.817	19	185.019	338.373	20	195.000	345.243
21	212.038	436.401	22	217.021	679.590	23	244.998	1141.266	24	255.025	580.626
25	272.046	462.383	26	309.012	339.526	27	312.022	281.534	28	342.010	409.552
29	409.072	928.865	30	426.083	1003.294	31	456.051	529.935	32	525.119	444.739
33	553.116	624.473	34	573.151	680.451	35	670.160	624.386	36	700.186	594.337
37	784.269	622.447	38	864.345	2595.671	39	881.368	1575.104	40	912.849	773.338
41	915.007	703.268	42	917.600	1160.140	43	920.027	952.770	44	928.792	2525.105
45	931.887	954.333	46	934.964	1026.075	47	947.885	732.689	48	951.527	803.564
49	954.384	588.801	50	961.343	1588.840	51	967.320	936.800	52	970.465	1046.126
53	972.593	724.868	54	975.926	582.192	55	978.339	1717.524	56	1042.630	538.341
57	1048.135	478.042	58	1056.134	527.660	59	1059.188	512.303	60	1064.440	869.548
61	1067.651	883.875	62	1071.014	1437.204	63	1074.114	1973.800	64	1078.194	734.161
65	1080.314	556.774	66	1082.548	874.114	67	1085.796	684.976	68	1125.483	1078.260

Calculated Masses:

FPPPNFPGPR

N-Term.	Ion	a	a-17	a-18	b	b-17	b-18	b+18	c	i	x	y	z	C-Term.	Ion
1	F	**120.081**	103.054	102.070	148.076	131.049	130.065	166.086	165.102	**120.081**	201.098	**175.119**	158.092	10	R
2	P	**217.134**	200.107	199.123	**245.128**	228.102	227.118	263.139	262.155	**70.065**	298.151	**272.172**	**255.145**	9	P
3	P	314.186	297.160	296.176	**342.181**	325.155	324.171	360.192	359.208	**70.065**	355.172	329.193	312.167	8	G
4	P	411.239	394.213	393.229	439.234	422.207	421.223	457.245	**456.261**	**70.065**	452.225	**426.246**	**409.219**	7	P
5	N	**525.282**	508.255	507.271	**553.277**	536.250	535.266	571.287	570.303	87.055	599.294	**573.314**	556.288	6	F
6	F	672.350	655.324	654.340	**700.345**	683.319	682.335	718.356	717.372	**120.081**	713.337	687.357	**670.331**	5	N
7	P	769.403	752.377	751.393	797.398	780.372	779.388	815.409	814.425	**70.065**	810.389	**784.410**	767.383	4	P
8	G	826.425	809.398	808.414	854.420	837.393	836.409	872.430	871.446	30.034	907.442	**881.463**	**864.436**	3	P
9	P	923.477	906.451	905.467	**951.472**	934.446	933.462	969.483	968.499	**70.065**	1004.495	**978.516**	**961.489**	2	P
10	R	1079.579	1062.552	1061.568	1107.573	1090.547	1089.563	**1125.584**	1124.600	**129.113**	1151.563	**1125.584**	1108.557	1	F

Figure S16. The LIFT spectrum analysis report for protegrin-4 precursor peptide FPPPNFPGPR. BioTools 3.2 was used for analysis.

MALDI MSI mean mass spectra

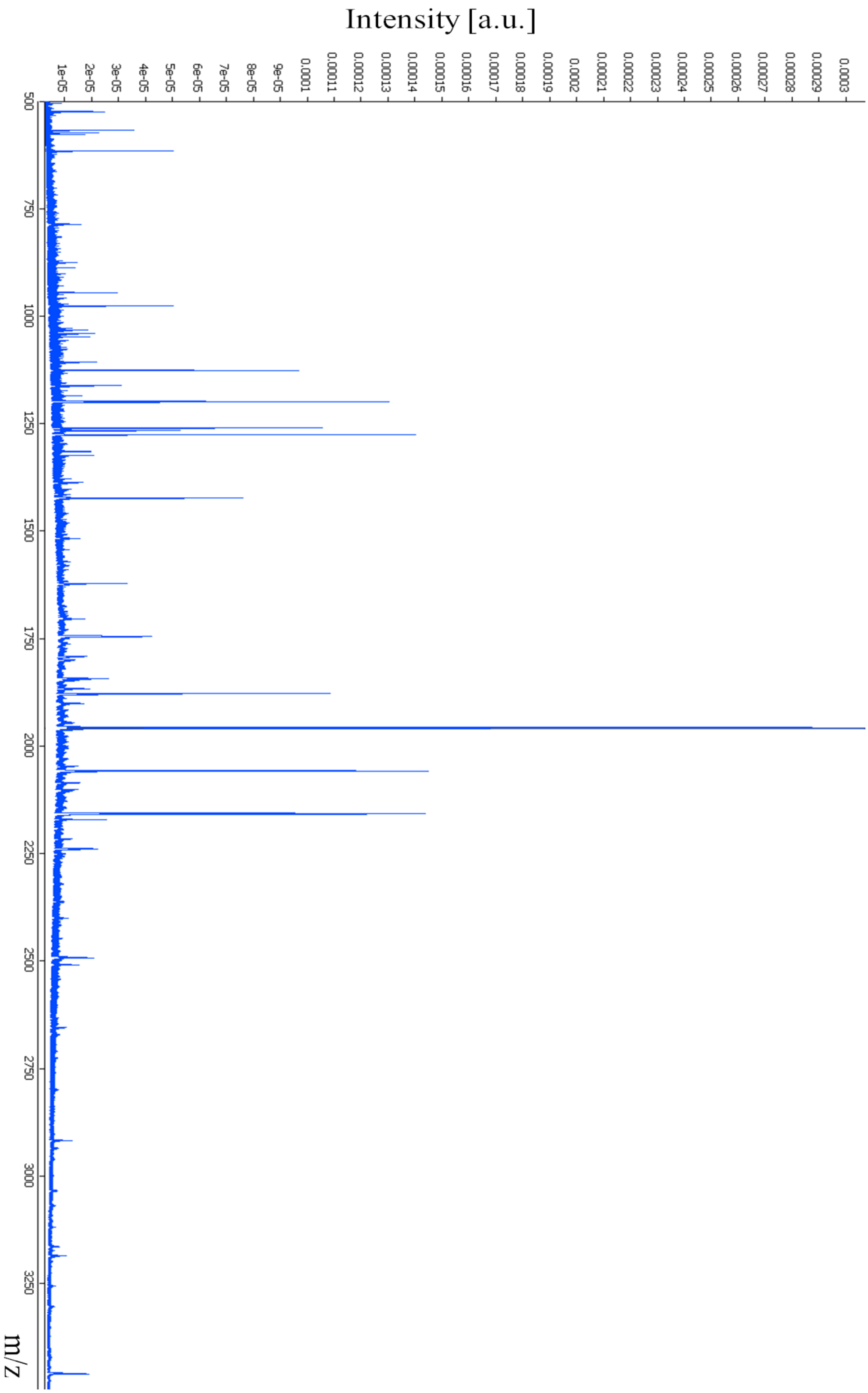
Intensity [a.u.]
1e-05
2e-05
3e-05
4e-05
5e-05
6e-05
7e-05
8e-05
9e-05
0.0001
0.00011
0.00012
0.00013
0.00014
0.00015
0.00016
0.00017
0.00018
0.00019
0.0002
0.00021
0.00022
0.00023
0.00024
0.00025
0.00026
0.00027
0.00028
0.00029
0.0003
500
750
1000
1250
1500
1750
2000
2250
2500
2750
3000
3250
m/z

Figure S17. The MALDI MSI mean mass spectrum created from all measured tissue sections. SCiLS Lab 2014b was used for processing the spectra applying baseline removal and normalization.

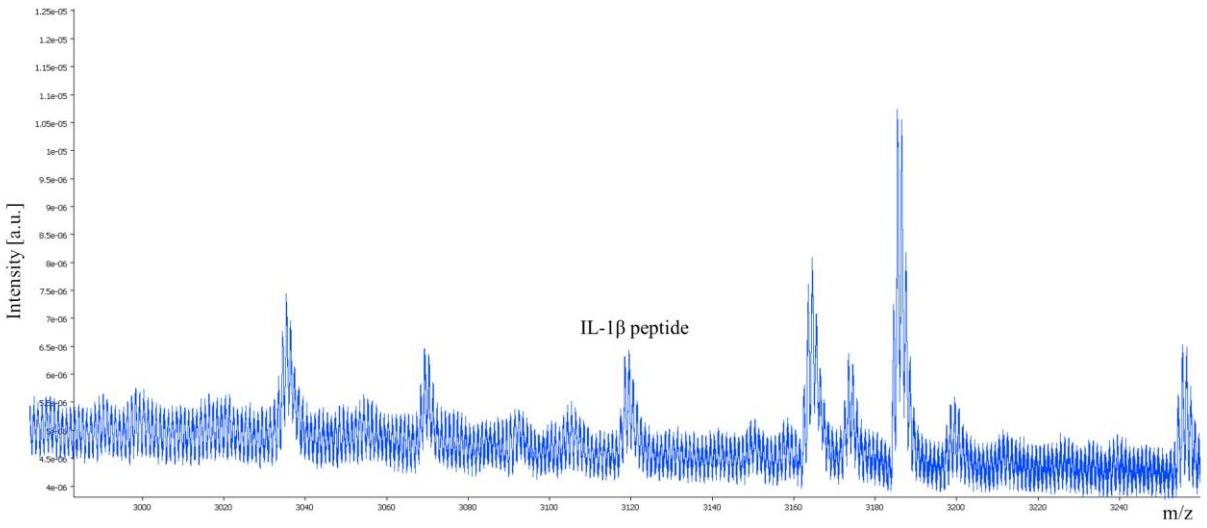

Figure S18. Zoomed MALDI MSI mean mass spectrum created from all measured tissue sections showing the peak of IL-1β peptide NLYLSCVLKDDKPTLQLESVDPKNYPK. SCiLS Lab 2014b was used for processing the spectra applying baseline removal and normalization.

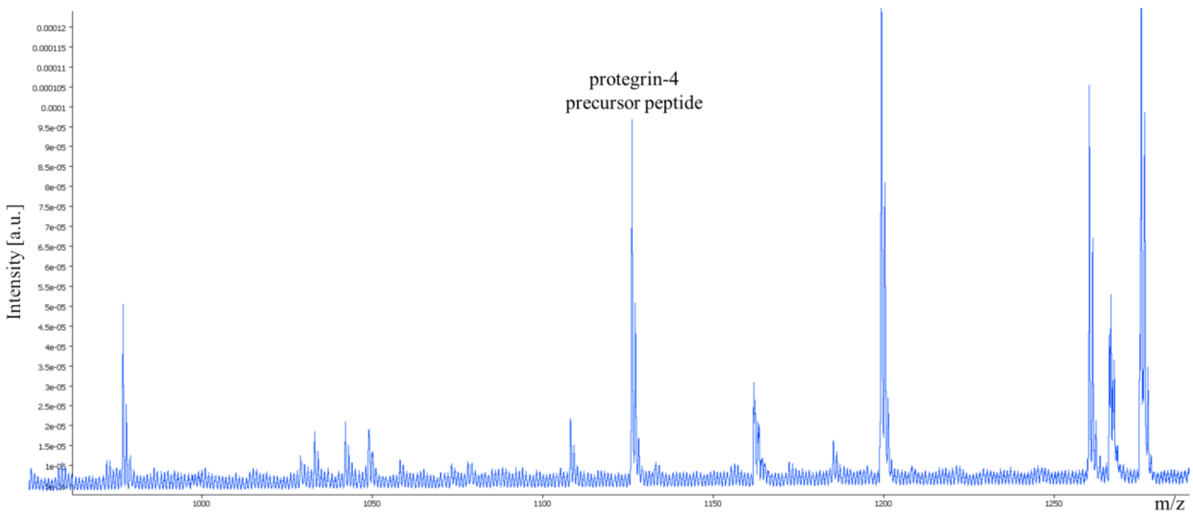

Figure S19. Zoomed MALDI MSI mean mass spectrum created from all measured tissue sections showing the peak of protegrin-4 precursor peptide FPPPNFPGPR. SCiLS Lab 2014b was used for processing the spectra applying baseline removal and normalization.

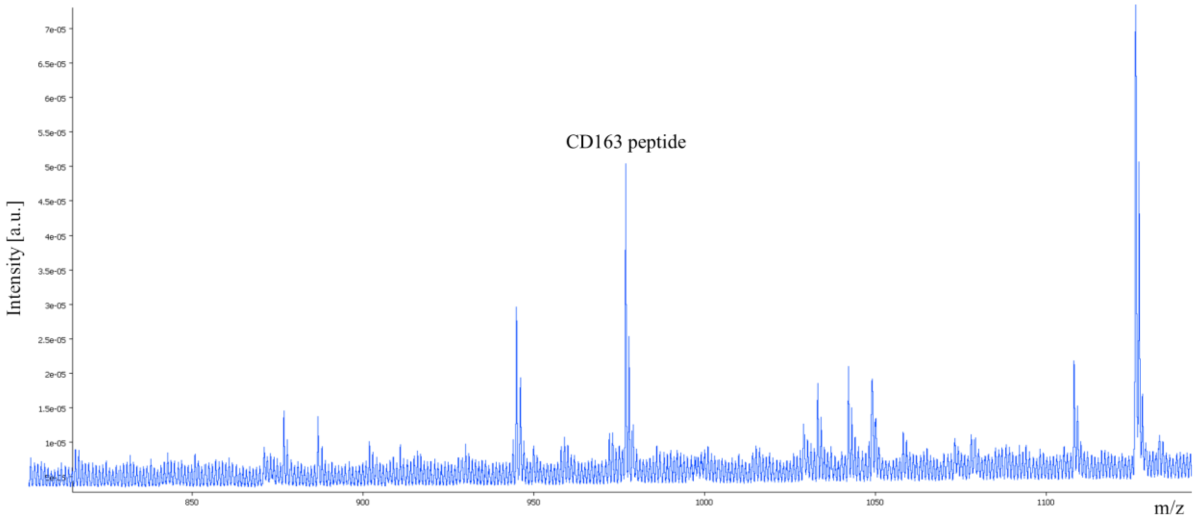

Figure S20. Zoomed MALDI MSI mean mass spectrum created from all measured tissue sections showing the peak of CD163 peptide TSYQVYSK. SCiLS Lab 2014b was used for processing the spectra applying baseline removal and normalization.

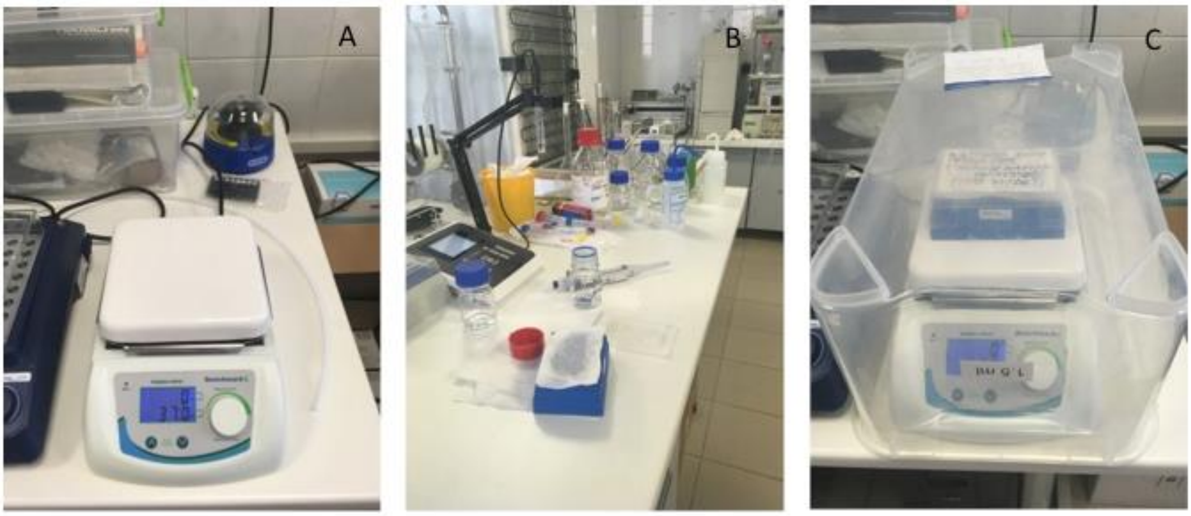

Figure S21. The preparation of humid box used for *on-tissue* trypsinization. (**A**) A magnetic stirrer with hotplate used for heating the humid box. (**B**) A humid box prepared from used tip rack, which was filled with water and on top was placed Kimwipe tissue soaked in LC-MS water. The ITO glass slides with applied trypsin solution were placed on the soaked Kimwipe tissue and the humid box/tip rack was closed. (**C**) A final set up used for 18h trypsinization.
